# RNA-Dependent Regulation of Virulence in Pathogenic Bacteria

**DOI:** 10.3389/fcimb.2019.00337

**Published:** 2019-10-09

**Authors:** Shubham Chakravarty, Eric Massé

**Affiliations:** RNA Group, Department of Biochemistry, Faculty of Medicine and Health Sciences, CRCHUS, University of Sherbrooke, Sherbrooke, QC, Canada

**Keywords:** regulatory RNA, riboregulation, UPEC, *S. aureus*, *L. monocytogenes*, *H. pylori*, *P. aeruginosa*

## Abstract

During infection, bacterial pathogens successfully sense, respond and adapt to a myriad of harsh environments presented by the mammalian host. This exquisite level of adaptation requires a robust modulation of their physiological and metabolic features. Additionally, virulence determinants, which include host invasion, colonization and survival despite the host's immune responses and antimicrobial therapy, must be optimally orchestrated by the pathogen at all times during infection. This can only be achieved by tight coordination of gene expression. A large body of evidence implicate the prolific roles played by bacterial regulatory RNAs in mediating gene expression both at the transcriptional and post-transcriptional levels. This review describes mechanistic and regulatory aspects of bacterial regulatory RNAs and highlights how these molecules increase virulence efficiency in human pathogens. As illustrative examples, *Staphylococcus aureus, Listeria monocytogenes*, the uropathogenic strain of *Escherichia coli, Helicobacter pylori*, and *Pseudomonas aeruginosa* have been selected.

## Introduction

Numerous bacterial species are infamous for their role in causing human diseases (Kusters et al., 2006; Gellatly and Hancock, 2013; Dayan et al., 2016; Terlizzi et al., 2017; Radoshevich and Cossart, 2018). These bacterial pathogens possess certain key distinguishing features. First, they can efficiently sense environmental cues presented by the host such as changes in nutrient availability, pH, osmolarity, and temperature (Fang et al., 2016). Second, pathogenic organisms quickly adapt their metabolic physiology accordingly, thereby switching between their free-living lifestyles and that within the host (Groisman and Mouslim, 2006; Fuchs et al., 2012). Finally, they are characterized by an arsenal of virulence attributes, which they robustly modulate to survive and proliferate during host infection (Pettersson et al., 1996). For example, bacteria harbor potent toxins and toxin delivery systems (Green and Mecsas, 2016). One major function of bacterial toxicity is to kill surveilling immune cells such as neutrophils (do Vale et al., 2016). While this is an immune-evading mechanism employed by pathogenic bacteria, the proteins constituting these toxins and their delivery conduits are highly immunostimulatory (Miao et al., 2010; Gall-Mas et al., 2018). Additionally, toxin expression and secretion are energy intensive (Lee and Rietsch, 2015; Joo et al., 2016). Thus, pathogenic bacteria cannot afford to constitutively express toxin genes but must modulate their expression according to the site and stage of the infection.

A second important virulence property of pathogenic organisms is their ability to form biofilms. A biofilm lifestyle, as markedly opposed to a single organismic one, is characterized by bacteria clustered with each other and attached to a foreign surface such as the host epithelium (Costerton et al., 1995). Another important feature includes encapsulation of the bacterial community inside of an extracellular matrix consisting of polymeric substances synthesized by the bacteria themselves such as polysaccharides (Sutherland, 2001). This abiotic outer layer adopts distinct three-dimensional structures. For example, it can form water channels, critical for efficient nutrient mobilization and uptake by biofilm bacteria (Stewart, 2003; Wilking et al., 2013). Additionally, this protective matrix provides a barrier against host immune responses (Leid et al., 2005; Begun et al., 2007; Toska et al., 2018; Tseng et al., 2018) and antimicrobial therapy (Goltermann and Tolker-Nielsen, 2017; Hall and Mah, 2017; Singh et al., 2017). Because of this, and due to altered gene expression, biofilm-associated bacteria are highly recalcitrant to antibiotics (Mah and O'Toole, 2001; Stewart, 2002; Hall and Mah, 2017). Consequently, bacterial biofilm formation represent a huge clinical burden, being widely implicated in the establishment and maintenance of chronic infections (James et al., 2008; Calo et al., 2011; Chen and Wen, 2011; Omar et al., 2017). A classic example is the formation of highly antibiotic resistant biofilms in the airways of Cystic Fibrosis (CF) patients (Lopez-Causape et al., 2015) (described later in detail). Other clinically relevant biofilm infections include otitis media (Bakaletz, 2007), and biofilms frequently found on medical devices such as catheters dwelling inside the patient (Donlan, 2008).

Another community-associated behavior contributing to bacterial virulence is quorum sensing (QS) (Antunes et al., 2010). QS is a bacterial cell-to-cell communication mechanism dependent on the abundance of signaling molecules, known as autoinducers (AI), in the extracellular milieu (Miller and Bassler, 2001). AIs are regulators of bacterial gene expression (Rutherford and Bassler, 2012; Banerjee and Ray, 2016, 2017). Each bacterial cell is capable of synthesizing and secreting AI molecules. Thus, the magnitude of AI accumulation hinges upon both bacterial cell-density as well as gene expression profile (i.e., whether the AI production is on or off) of the whole bacterial community. At an adequate cell density, when the AI levels reach a certain threshold, they are detected by receptors located in the bacterial cell membrane or in the cytoplasm. Some of these receptors comprise the membrane-associated sensor histidine kinase of bacterial two-component signal transduction systems. Binding of the AI to the receptor activates its kinase activity thus autophosphorylating it, followed by transmission of the phosphate group to the corresponding response regulator, thereby facilitating regulation of genes in that particular QS regulon. The second mechanism of QS mediated regulation starts with secretion of the inactive AI. In the extracellular environment, it is processed to its active form, and either diffuses freely or is transported back into bacterial cells. There, the AI binds its cognate cytoplasmic receptor, which is characteristically a global transcription factor controlling the whole QS regulon (Rutherford and Bassler, 2012).

**Figure 1:**
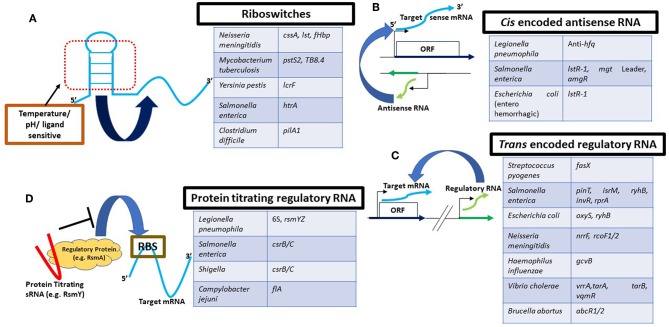
Schematic representation of major types of RNA-based regulatory mechanisms in pathogenic bacteria. **(A)** Riboswitches are most commonly part of the 5′UTR of the corresponding target mRNA. They are responsive to chemical ligands or environmental signals such as temperature (as in an RNA thermometer) for structural rearrangements leading to gene expression changes. sRNAs can be expressed from the complimentary strand **(B)** (as antisense RNA) or from a different genomic location **(C)**. Blue: transcript under regulation; green: regulatory RNA. **(D)** Regulatory proteins (yellow) typically bind their target mRNAs (blue) at the RBS or the SD sequence to modulate their stability and/or activate/inhibit translation. Protein binding sRNAs (red) on the other hand can sequester these regulatory proteins by direct binding and titrate them away from their targetome. Pathogenic organisms not described in the main text are referred in this figure to highlight different RNAs as examples. For more details, refer to the following articles that have been extensively referred to for construction of this figure (Svensson and Sharma, 2016; Westermann, 2018).

Altered gene expression is key to a pathogen's optimization of its virulence attributes. For example, significant changes exist in both transcript and proteome profiles of the same bacterial species existing as a free-floating single bacterium vs. in a biofilm (Oosthuizen et al., 2002; Nigaud et al., 2010; Chavez-Dozal et al., 2015; Charlebois et al., 2016; Jia et al., 2017; Favre et al., 2018). Bacterial regulatory RNAs are now established as pivotal players in facilitating these coordinated changes in gene expression, acting at all levels, starting from transcription to protein translation and protein activity (Romby et al., 2006; Toledo-Arana et al., 2007; Svensson and Sharma, 2016; Westermann, 2018). These RNA regulators can be classified in different groups, as detailed below (Figure 1).

The first type of regulatory RNA elements includes those present in the 5′ untranslated regions (UTR) of their cognate mRNA. 5′UTRs can contain complex structures that undergo alterations depending on environmental conditions (Winkler and Breaker, 2005; Waters and Storz, 2009). These 5′UTR structure are known as riboswitches and are important regulators of gene expression at the transcriptional or the translational level. Riboswitches respond to changes in abundance of small metal ions, small molecules, or metabolites (Winkler and Breaker, 2005; Waters and Storz, 2009). Another example of 5′UTR regulatory elements are RNA thermometers, which responds to temperature changes during infection (Loh et al., 2018). For instance, the start codon of an mRNA might be embedded in the 5′UTR region, which adopts a stem loop structure at lower temperatures, thus preventing translation. Upon colonization of the host, this stem loop loosens as the temperature increases (>37°C), facilitating translation. Finally, another type of 5′UTR regulatory elements respond to pH changes, by forming inaccessible structures at one pH and opening up at a different one (Nechooshtan et al., 2009).

The second type of regulatory RNAs are encoded in *cis* and are known as anti-sense RNAs (asRNA). They regulate (i) transcription efficiency, by transcription interference, (ii) RNA stability, by forming RNA-RNA double stranded complex which may be degraded, and (iii) translation initiation, by interacting with and sequestering the ribosome binding site (RBS) (Svensson and Sharma, 2016; Westermann, 2018).

The third type of regulatory RNAs are expressed in *trans*, that is, at a different genomic site than the genes they regulate (Svensson and Sharma, 2016; Westermann, 2018). They are known as small regulatory RNAs (sRNA). The first mechanism employed by sRNAs involves binding of the sRNA to a regulatory protein to titrate it away from its target. For example, the 6S RNA is expressed upon entry in stationary phase of growth and acts by titrating the RNA polymerase holoenzyme containing the sigma70 (σ^70^) specificity factor, preventing transcription dependent on σ^70^ (Wassarman, 2007). The second mechanism employed by sRNAs involves direct RNA-RNA base-pairing of the sRNA to its target mRNA. In instances of inhibition of gene expression, the sRNA base-pairs with the RBS of the target mRNA, thus occluding translation. In cases of positive regulation, the sRNA have been reported to act by binding to sequences in the 5′UTR of the target mRNAs and preventing the formation of inactivating stem-loop structures (Morfeldt et al., 1995).

Additionally, proteins commonly interact with the regulatory RNAs mentioned above in facilitating the observed regulatory roles. For example, the hybridization of sRNAs with their target mRNAs in Gram negative bacteria is often mediated by the RNA chaperone Hfq (Updegrove et al., 2016). Furthermore, downstream degradation of mRNAs targeted by sRNAs is mostly attributed to RNases (Matos et al., 2017).

Despite different types of bacterial regulatory RNAs and mechanisms of action, certain paradigms can be deduced (Westermann, 2018). One striking feature is that while some regulatory RNAs do have specific stray targets, they mostly target the mRNA encoding a central regulatory player such as a global transcription factor, thereby acting on a large number of indirect targets at once. This determines major virulence transitions such as switching over from a planktonic lifestyle to a biofilm lifestyle (Williams McMackin et al., 2019). Secondly, in multiple pathogenic bacteria, reshaping of metabolism, and virulence by the action of regulatory RNAs appear to be intertwined. A prominent example is the sugar-phosphate stress (thus related to metabolism) associated sRNA SgrS, which also regulates the pathogenesis effector SopD (Papenfort and Vogel, 2014) in *Salmonella*. A second example is the TarA sRNA in *Vibrio*, which links virulence with glucose acquisition (Richard et al., 2010). Finally, it appears that functional redundancy among multiple regulatory RNAs exists, with more than one of them facilitating the same regulation, albeit to different intensities (Deng et al., 2012, 2014; Heidrich et al., 2017; Pannekoek et al., 2017).

In light of the major virulence attributes and RNA based regulatory mechanisms described previously, this review aims to describe specific pathways of riboregulation of virulence factors in prominent human pathogens (Table 1). For this purpose, we will focus on pathogens affecting different niches of infection; *Staphylococcus aureus* for disseminated systemic infections and those associated with prosthetic implants; *Listeria monocytogenes* as a model intracellular bacterial pathogen; UPEC (uropathogenic *Escherichia coli*) for urinary tract infections; *Helicobacter pylori* as an enteric pathogen and *Pseudomonas aeruginosa* as a major causative agent of airway infections in cystic fibrosis patients.

**Table 1:** List of riboregulatory molecules described in the text.

**Pathogen**	**Regulatory** **RNA/Protein**	**Mechanism of Action**
*Staphylococcus aureus*	RNAIII	Trans acting sRNA
	RsaA	Trans acting sRNA
	SprD	Trans acting sRNA
*Listeria monocytogenes*	Rli27	Trans acting sRNA
	LhrC	Trans acting sRNA
	Rli55	Riboswitch
	AspocR	Riboswitch
	PfrA	Riboswitch
	SreA	Riboswitch
	SreB	Riboswitch
	Anti0677	Antisense RNA (Excludon)
UPEC	PapR	Trans acting sRNA
	RyhB	Trans acting sRNA
*Helicobacter pylori*	IsoA1	Trans acting sRNA
	RepG	Trans acting sRNA
	5′*ureB*	Trans acting sRNA
*Pseudomonas aeruginosa*	RsmA	RNA binding protein
	RsmF	RNA binding protein
	RsmV	Trans acting sRNA
	RsmW	Trans acting sRNA
	RsmY	Trans acting sRNA
	RsmZ	Trans acting sRNA
	CrcZ	Trans acting sRNA
	ReaL	Trans acting sRNA
	PhrS	Trans acting sRNA
	PrrF1	Trans acting sRNA
	PrrF2	Trans acting sRNA
	ErsA	Trans acting sRNA
	Sr0161	Trans acting sRNA

## Staphylococcus aureus

The Gram-positive bacteria *S. aureus* is often present among the normal human skin microbiome (Becker and Bubeck Wardenburg, 2015). However, it is also one of the most common pathogens implicated in bacterial infections of all areas of the body including skin (McCaig et al., 2006), bones (Olson and Horswill, 2013), heart (Fernandez Guerrero et al., 2009), respiratory tract (Parker and Prince, 2012), and bloodstream (Corey, 2009). Additionally, it is well-known to form highly persistent biofilms on prosthetic devices and implants (Lister and Horswill, 2014). *S. aureus* is one of the primary causative agents of nosocomial infections, a majority of which are antibiotic resistant (Wang and Ruan, 2017). This pathogen is listed in the ESKAPE (*Enterococcus faecium, Staphylococcus aureus, Klebsiella pneumonia, Acinetobacter baumannii, Pseudomonas aeruginosa*, and *Enterobacter* sp.) group of bacteria, which represent the most antibiotic resistant species (Santajit and Indrawattana, 2016). *S. aureus* possesses a myriad of virulence mechanisms including expression of toxins, surface adhesins, immune-evading molecules, quorum sensing, and biofilm formation (Powers and Bubeck Wardenburg, 2014). These pathogenic determinants are intricately regulated, and sRNAs play a significant role in that regulatory network (Fechter et al., 2014; Tomasini et al., 2014). Key sRNAs of *S. aureus* are described below.

### RNAIII

The best characterized sRNA in *S. aureus* is RNAIII (Boisset et al., 2007; Bronesky et al., 2016). RNAIII is under control of the *agr* QS system. The *agr* locus comprises of two ORFs (open reading frames), transcribed by promoters P2 and P3 in opposite directions. P2 drives the transcription of a four-cistron mRNA, RNAII. Among these four gene products, AgrD is an autoinducer peptide (AIP) synthesized in its inactive form. AgrB is a membrane associated AIP transporter, which matures the precursor AgrD AIP to its active form and exports it out of the cell. The remaining two cistrons *agrC* and *agrA* form the sensor histidine kinase and its cognate response regulator, respectively, in a two-component signaling (TCS) cascade. At high cell density, the autoinducer peptide AgrD is detected by the sensor AgrC and the signal is globally transmitted intracellularly by the now phosphorylated response regulator, AgrA. AgrA, in turns, upregulates transcription of RNAIII (from promoter P3) that will exert pleiotropic roles in *S. aureus*.

First, the 5′ region of RNAIII encodes the δ-hemolysin, conferring hemolytic activity to the bacterium. Then, RNAIII can act as a regulatory RNA, regulating multiple target mRNAs. RNAIII has a long half-life of 45 min and is structurally characterized by 14 stem-loops and two lengthy helical structures. Specific hairpins are involved in base-pairing to target mRNAs, with more than one stem-loop acting in concert to achieve regulation. To repress translation, RNAIII may bind at the RBS (e.g., *lytM* mRNA), both at the RBS and the 5′UTR (e.g., *rot* mRNA), using multiple stem loops, or at the coding region (e.g., *coa* mRNA) (Felden et al., 2011). RNAIII can also positively regulate targets. The only two targets known to be upregulated by RNAIII are *hla* mRNA, encoding the α-hemolysin, and *eap*, encoding the extracellular adherence protein (Guillet et al., 2013).

While it has numerous mRNA targets to which it directly binds, RNAIII also modulates a large indirect regulon, for example, by inhibiting translation of the key transcriptional repressor *rot* (*r*epressor *o*f *t*oxins) (Geisinger et al., 2006). Of note, as in most cases of regulatory RNA binding events in Gram-positive pathogens, RNAIII does not require the RNA chaperone Hfq, even though it has been shown to bind to RNAIII *in vitro* (Liu et al., 2010).

The overarching feature of RNAIII-mediated regulation is that it represses translation of genes encoding for surface proteins or those associated with high peptidoglycan turnover, which are typically required at primitive stages of infection marked by low cell numbers to facilitate and consolidate early events in bacterial colonization. Conversely, it activates synthesis of secreted exotoxins, which are required for bacterial dissemination at later time points of infection when bacterial cell density is high. Indeed, RNAIII is reported to assist *S. aureus* switch from a biofilm mode of growth (colonization and persistence in a new niche) to a more invasive one, required for dispersal to new host tissues (Boisset et al., 2007). Observations that *S. aureus* isolates from antibiotic resistant chronic bacteremia, like those associated with prosthetic implants, are commonly defective in both *agr* locus and RNAIII expression, further bolster this view. RNAIII holds a pivotal position in *S. aureus* regulation of virulence. Evidently, apart from a few exceptions, all downstream effects of *S. aureus* QS signaling are mediated through RNAIII. RNAIII is therefore versatile as a regulator, cascading both direct and indirect pathways.

Methicillin resistant *S. aureus* (MRSA) are considered the most dangerous of *S. aureus* isolates. The mobile genetic element SCCmec was shown to confer resistance to methicillin (Noto et al., 2008). Interestingly, the mRNA of one of the genes in this region, *psm-mec*, binds to and inhibits translation of the previously described *agrA* gene. Consistent with reports that the *agr* system and its effectors, such as RNAIII, produce a pronounced invasive character in *S. aureus, psm-mec* mutations in community-acquired MRSA isolates account for their acute virulence nature (Qin et al., 2016).

### RsaA

Contrarily to RNAIII, the sRNA RsaA promotes chronic persistence, biofilm formation, and expression of cell surface proteins (Romilly et al., 2014). The *rsaA* gene is under positive transcriptional control of the specialized factor SigmaB (σ^B^) and is minimally expressed at exponential phase and highly expressed at stationary phase (Geissmann et al., 2009). Furthermore, both endoribonucleases RNase III and RNase Y regulate its degradation (Romilly et al., 2014). The primary target of RsaA is the *mgrA* mRNA, encoding the global transcriptional regulator MgrA, whose translation is inhibited by base-pairing of the sRNA. Rate constant for this binding indicates rapid association, which is important for impeding the formation of ribosomal initiation complex (Romilly et al., 2014). The translationally repressed *mgrA* mRNA is then likely degraded. RsaA can bind to two distinct regions of the *mgrA* mRNA: a cytosine-rich motif targets the Shine-Dalgarno (SD) and two hairpin loops in the 5′ region of RsaA interact with the coding sequence of *mgrA*.

MgrA has ~350 genes in its regulon, including its own mRNA that is positively autoregulated (Luong et al., 2006). The culminating effect of MgrA is activation of capsule synthesis and inhibition of biofilm formation by repressing surface proteins expression and releasing extracellular DNA (Trotonda et al., 2008). Intriguingly, through *mgrA* modulation, RsaA is connected with RNAIII and the *agr* QS system, as MgrA activates transcription of the *agr* locus (Ingavale et al., 2005).

It is postulated that presence of a functional RsaA may have been evolutionarily favored in *S. aureus*. Given that it is primarily a commensal organism in the human host, it is possible that RsaA prevents the bacteria from being hyperinvasive at all times, and this regulation is critical for normal colonization fitness (Romilly et al., 2014). Using MAPS (a technology developed to affinity purify RNA-RNA complexes *in vivo* and identify the targets by sequencing), a recent study has further validated the interaction of *mgrA* mRNA with RsaA and has further extended its RNA targetome (Tomasini et al., 2017).

### SprD

SprD is a sRNA transcribed from a pathogenicity island (region on the chromosome predominately harboring virulence associated genes) whose main target elucidated thus far is the *sbi* mRNA, encoding the immune evading effector Sbi (Second binder of *i*mmunoglobulins) (Chabelskaya et al., 2010). Sbi inhibits opsonization, which is usually followed by phagocytosis and action of the complement system (Haupt et al., 2008). Specifically, Sbi binds IgG, the C3b, and H complement factors for these purposes. While these functions of Sbi are important in the survival of *S. aureus* in the host, it is to be noted that Sbi nevertheless elicits a major pro-inflammatory response by activating multiple immune-signaling cascades that results in the production of major cytokines (IL-6 for example) and leukocytes chemotaxis to the site of infection. Thus, for success of the organism, Sbi expression needs to be optimally fine-tuned rather than constitutively expressed. The regulatory RNA SprD facilitates this by keeping Sbi levels in check through impeding its translation. Indeed, part of the 5′UTR, the SD sequence and the start codon of *sbi* have been reported to be critical for the regulation by SprD. Consistent with that seen with RNAIII, SprD does not require the RNA chaperone Hfq in binding its target. Though blocking translation of *sbi*, SprD does not facilitate degradation of the mRNA. The fact that SprD is a major regulator of *S. aureus* pathogenesis is evident from the fact that its deletion severely decreases morbidity and mortality of the mouse model of infection. Interestingly, RNAIII also binds to and represses *sbi* translation by a similar mechanism, further illustrating the need for only modest synthesis of the Sbi protein.

## Listeria monocytogenes

*L. monocytogenes* is an especially interesting model to describe the role of regulatory RNAs in shaping pathogenesis during the intracellular lifestyle of a bacterium. In addition, *L. monocytogenes* represents a group of uncommon bacterial pathogens. Indeed, in contrast to other pathogens described in this review, *L. monocytogenes* infections (listeriosis) are relatively scarce. Nevertheless, listeriosis mortality rates remain in the high range of 20–30% (Radoshevich and Cossart, 2018), particularly for children, the elderly, immunocompromised patients, and during pregnancy. The primary mode of transmission is via contaminated food, and for people in vulnerable groups, the minimum infective dose can be as low as 100 bacteria (Radoshevich and Cossart, 2018). As a gastro-intestinal pathogen, this bacterium breaches the intestinal epithelial barrier and spreads via blood and lymphatic system to the whole body, colonizing especially the liver and spleen (Cossart, 2011). Importantly, it resides intracellularly in both phagocytic and non-phagocytic cells, helped by an arsenal of virulence factors modulating host cell processes (Pizarro-Cerda et al., 2012). Higher than 150 sRNAs have been reported in *L. monocytogenes* (Lebreton and Cossart, 2016). Some of those involved in intracellular pathogenesis are described in this section.

### Rli27

Lm0514 is a protein in *L. monocytogenes* that is recognized by sortase enzymes through its LPXTG motif and is tethered to peptidoglycan located on the cell surface (Garcia-del Portillo et al., 2011; Mariscotti et al., 2012). Importantly, Lm0514 protein level is highly augmented during intracellular growth of the bacteria, where it significantly facilitates survival (Pucciarelli et al., 2005; Garcia-del Portillo et al., 2011). Interestingly, *lm0514* mRNA level increases only by 6-fold during intracellular vs. extracellular growth, while the rise in protein level is 200 times higher (Garcia-del Portillo et al., 2011). This is strongly indicative of posttranscriptional regulation, and indeed, during infection, the *lm0514* mRNA transcript is upregulated by the sRNA Rli27. Rli27 binds to the 5′UTR of *lm0514*, exposing the RBS and enhancing translation (Quereda et al., 2014).

### LhrC

*Listeria* Hfq-binding RNA C (LhrC) is a small non-coding (nc) RNA first discovered amongst a pool of RNAs that co-immunoprecipitated with the RNA chaperone Hfq in *L. monocytogenes* (Christiansen et al., 2006; Sievers et al., 2014). However, later studies have established that, as in most cases of RNA based regulation in Gram-positive species, Hfq is not required for LhrC stability or its interaction with mRNA targets. There are five copies of the *lhrC gene* ranging from 111 to 114 nucleotides in size (Sievers et al., 2014). Initial observations of LhrC expression during *L. monocytogenes* intracellular growth in macrophages and its putative role in facilitating pathogenesis have been substantiated by three reports of Kallipolitis and colleagues (Sievers et al., 2014; Dos Santos et al., 2018; Ross et al., 2019). LhrC RNAs 1-5 (LhrC1-5) were found to be upregulated during conditions of cell envelope stress such as those generated by the antibiotic cephalosporin or bile salts (Sievers et al., 2014). A later study reported that heme, commonly encountered by *L. monocytogenes* during systemic dissemination in human hosts, is also a trigger for LhrC expression (Dos Santos et al., 2018).

The first LhrC target characterized was the mRNA *lapB*, encoding a cell wall-tethered adhesin (Sievers et al., 2014). LhrC contains three regions with high concentration of cytosine, resulting in a UCCC motif which is critical in mediating interaction with the AG rich SD sequence of the target mRNA to repress translation. Downregulation of *lapB* expression probably benefits the organism in evading host immune response while spreading through blood, as bacterial surface proteins are well-known immune stimulators (Toledo-Arana et al., 2009). Five copies of LhrC and three redundant CU-rich target binding sites indicate the need and the potential of LhrC to transform a low input signal into a magnified output response. Further, the five copies of LhrC appear to act additively rather than redundantly, corroborating this hypothesis. Subsequent work has established that LhrC, by using its UCCC motifs, binds to and represses mRNAs of genes involved in heme influx into the cell and its subsequent metabolism (Dos Santos et al., 2018). Thus, it is not surprising that LhrC plays an important role in survival of the pathogen in heme-rich niche containing lysed erythrocytes.

By using the same motif and mechanism, LhrC also represses mRNA translation of another membrane protein-coding mRNA, *oppA*, involved in oligopeptide binding (Sievers et al., 2015). Recent work has demonstrated the inhibitory mechanism of LhrC on another mRNA target, the T cell stimulating antigen (TcsA) (Ross et al., 2019). Here, the repressive mechanism involves LhrC base pairing at a site upstream of the SD region of *tcsA*, which does not affect translation but rather induces rapid transcript turnover.

### Riboswitch-Regulated Nutrient Utilization

During gastro-intestinal infection in vertebrate hosts, *L. monocytogenes* commonly encounters ethanolamine, and metabolizes it by expressing the *eut* (*e*thanolamine *ut*ilization) genetic locus (Garsin, 2010; Archambaud et al., 2012). Transcription of *eut* is under RNA-based regulation (Freitag, 2009).

The first level of regulation is mediated by the sensor histidine kinase EutW and its cognate response regulator EutV, which are activated by presence of ethanolamine in the extracellular milieu. Phosphorylated EutV serves as an ANTAR antiterminator that prevents *eut* gene transcription cessation by binding to stem-loop structures in the nascent mRNA (Fox et al., 2009; Lebreton and Cossart, 2016). Located upstream of the *eut* locus is the gene Rli55, acting as the second level of RNA-based *eut* regulation. The 5′ region of Rli55 harbors a vitamin B12 riboswitch.

In absence of vitamin B12, Rli55 is transcribed as a 450 nt-long transcript, which sequesters EutV, thus leading to transcriptional attenuation of *eut* genes. Conversely, in presence of vitamin B12, the riboswitch binds its ligand, resulting in transcription of a much shorter Rli55 transcript (200 nt), incapable of sequestering EutV, leading to *eut* transcriptional antitermination and ultimately, *eut* expression (Mellin et al., 2014). Both ethanolamine (for EutV phosphorylation) and vitamin B12 (for Rli55 inhibition) are therefore essential to activate the ethanolamine utilization pathway in *L. monocytogenes*.

Another example where a vitamin B12 riboswitch controls nutrient utilization in *L. monocytogenes* is AspocR, which regulates propanediol usage by *L. monocytogenes* during infection (Mellin et al., 2013). Expression of the *pdu* (*p*ropane*d*iol *u*tilization) operon is driven by the transcription factor PocR (Kim et al., 2014). The complementary strand of *pocR* mRNA region encodes the riboswitch-controlled asRNA AspocR. In the absence of vitamin B12, this riboswitch serves as an antiterminator to the asRNA of *pocR* (AspocR), located downstream. Thus, under these conditions, the asRNA AspocR blocks expression of *pocR* and hence of *pdu* genes. In presence of vitamin B12, the riboswitch conformation promotes transcriptional attenuation of AspocR, and the resultant truncated asRNA is unable to bind the *pocR* mRNA transcript, allowing utilization of propanediol.

### Regulation of *PrfA* Expression

Translational control of *pfrA*, a master transcriptional activator of virulence genes in *L. monocytogenes* (de las Heras et al., 2011) is a notable example of RNA-based regulation. The temperature-sensitive thermoswitch located in its 5′UTR forms a hairpin at 30°C, thereby occluding the RBS and hindering *prfA* translation. Conversely, at the host infection temperature of 37°C, this secondary RNA structure is not favored resulting in upregulation of *prfA* translation. With PrfA activating expression of a myriad of toxins, lytic enzymes and actin-remodeling proteins, this mechanistic control ensures that virulence factors are produced by the bacteria when in the host (37°C) but not otherwise (30°C) (Lebreton and Cossart, 2016). A second control mechanism is associated with nutrient availability and is exerted by sRNAs SreA and SreB (Loh et al., 2009). In the presence of the ligand S-adenosylmethionine (SAM), premature transcriptional termination occurs in riboswitches SreA and SreB facilitating expression of smaller non-coding transcripts which bind to *prfA* mRNA RBS, preventing translation.

### Excludon-Mediated Control of Flagellar Motility

Excludon is a gene locus wherein the transcript serves as both an antisense RNA (asRNA) to block expression of the mRNA transcribed in the opposite direction as well as being the mRNA of adjacent gene(s) (Schultze et al., 2015; Lebreton and Cossart, 2016). Thus, an excludon negatively regulates its complementary gene but promotes expression of the neighboring gene in the same DNA strand. A classic example in *L. monocytogenes* is regulation of flagella biosynthesis, the cellular appendages facilitating bacterial swimming in liquid milieu and swarming on semisolid surfaces (Sesto et al., 2012). In *L. monocytogenes*, loci *lmo0675-0689* encodes the flagella-related *fli* operon. *lmo0676* and *lmo0677* encode proteins FliP and FliQ, which are integral parts of the flagellar export apparatus. On the opposite strand, a promoter drives the expression of a long RNA, named Anti0677, harboring full sequence complementarity to *lmo0675, lmo0676*, and *lmo0677*. Anti0677 acts as an antisense RNA, downregulating expression of the flagellar export apparatus. Additionally, transcription of Anti0677 also reads through *mogR*, the motility gene repressor. Anti0677 therefore acts as a mRNA, increasing MogR levels in the cell. This excludon regulates flagella synthesis from two angles: an asRNA mechanism leads to decrease in flagellar export apparatus and expression of *mogR* from two promoters (*anti0677* promoter and *mogR* promoter) increases MogR production, which also downregulates flagellar expression.

## Uropathogenic *Escherichia coli* (UPEC)

In the human host, pathogenic strains of *E. coli* cause infection in a plethora of sites, including the urinary tract (Kaper et al., 2004; Terlizzi et al., 2017). The uropathogenic *E. coli* (UPEC) is the primary cause of urinary tract infection (UTI), affecting both the urinary bladder (cystitis) and the kidney (nephritis), with widespread morbidity and even mortality. UPEC bacteria are armed with a variety of toxins, adhesins, and iron scavenging molecules called siderophores (Terlizzi et al., 2017). They also have excellent stress response systems and have the capability to form biofilms, even intracellularly (Anderson et al., 2003).

Regulators are essential determinants of UPEC virulence. The RNA chaperone Hfq has been reported to be important for UPEC colonization of mouse urinary tract (Kulesus et al., 2008). Intracellular microcolony formation, a hallmark of UPEC infections, as well as biofilm formation, which increases UPEC persistence, will not be as efficient upon Hfq deletion. Additionally, Hfq maintains lipopolysaccharide homeostasis, mediates tolerance to cell envelope stress, cationic antimicrobial polymyxin B, reactive free radicals and acidic conditions, on top of facilitating motility.

Commonly associated to the chaperone Hfq in *E. coli* are small regulatory RNAs. sRNAs play major roles in coordinating UPEC's virulence. Discussed below are the specific roles played by two important regulatory RNAs in this pathogenic bacterium.

### PapR

As described above, Hfq is a major regulator of virulence in UPEC. Because of its well-known RNA chaperone role, a group aimed at co-immunoprecipitating RNAs with Hfq to try and identify novel Hfq-associated sRNAs expressed during infection (Khandige et al., 2015). Hfq-bound sRNA profiles varied greatly depending on whether they were obtained from UPEC growing under lab conditions or within host cells. Particularly, envelope stress related sRNAs were found to increasingly co-precipitate during infection conditions.

The same screen uncovered the novel *trans* acting sRNA PapR, which negatively regulates *papI* mRNA, encoding a regulator of the adhesin P-fimbriae, a critical pathogenic factor aiding bacterial attachment to renal tissue (Lane and Mobley, 2007; Khandige et al., 2015). PapI is an activator of P-fimbriae biosynthesis, which turns on transcription of the P-fimbriae associated *pap* operon. PapR has been found to base-pair within the coding sequence of *papI* mRNA, ~80 nt downstream of its translational start site, to achieve negative translational regulation.

### RyhB

In non-pathogenic *E. coli*, the 90 nt-long sRNA RyhB regulates iron usage and uptake (Massé and Gottesman, 2002). Congruently, in UPEC, it promotes synthesis of iron-scavenging siderophores enterobactin, aerobactin, and salmochelin, critical for pathogenesis in the host environment (Porcheron et al., 2014). RyhB facilitates siderophore biosynthesis by base-pairing with mRNAs of the precursor molecules thereby stabilizing them and enhancing translation. Further supporting its role as a virulence mediator, in animal models of UTI, deletion of *ryhB* leads to defects in colonization of urinary bladder. RyhB also regulates infection in various other pathogens such as *Shigella* (Murphy and Payne, 2007) and *Vibrio* (Oglesby-Sherrouse and Murphy, 2013).

## Helicobacter pylori

*H. pylori* is an important pathogen using RNA-based virulence regulation to infect the gastric mucosa. Stomach of 50% of the total human race is believed to be colonized by this Gram-negative organism, which will remain in the gastric mucosa unless treated with persistent antimicrobial therapies (Testerman and Morris, 2014). Manifestations of *H. pylori* infections range from mild inflammation of the gastric tissue to severe and chronic peptic ulceration and finally to malignancies, the pathology with the worst prognosis (Wroblewski et al., 2010). *H. pylori* is known to modulate expression of micro RNAs (miRNAs) in the gastric tissue, altering the human immune response to its advantage (Libânio et al., 2015). This pathogen also possesses an array of other virulence factors, such as sRNAs, to survive and proliferate in the host (Pernitzsch and Sharma, 2012). *In silico* analysis has indeed revealed that multiple sRNAs might have important effects on its infectivity. Below are described three major *H. pylori* ncRNAs and their modes of action.

### IsoA1

Type 1 toxin-antitoxin systems are mechanistically represented by the mRNA of the toxin gene being inhibited by binding of the antitoxin sRNA (Unterholzner et al., 2013). In *H. pylori*, synthesis of the toxic 30 aa polypeptide AapA1 is stalled by base pairing of the IsoA1 sRNA, which serves as the corresponding antitoxin in this system (Arnion et al., 2017). During log phase growth, both *aapA1* and *isoA1* are constitutively transcribed. However, the 250 nt full length *aapA1* transcript is translationally inactive because of internal secondary structures occluding the RBS. It is only after the 3′end is processed, leading to a truncated 225 nt mRNA, that the RBS becomes available for translational initiation. However, this active structure also facilitates IsoA1 base-pairing, which leads to quick degradation of the sRNA-mRNA complex by RNase III. Thus, AapA1 toxin synthesis is repressed at two posttranscriptional levels; first by its own 5′UTR secondary structure, and second by the IsoA1 sRNA and RNase III, ensuring that the toxin is not formed during exponential growth of *H. pylori*.

### RepG

Implicated in *H. pylori* infections of animal models, TlpB is a chemotaxis receptor positively responding to quorum sensing signals and negatively responding to low pH (Croxen et al., 2006). *tlpB* mRNA has a characteristic 6–16 guanine repeat, termed simple sequence repeats (SSR), in its 5′ leader region. Variation in *tlpB* transcript SSR length is observed between *H. pylori* isolated from different patients, and sometimes even from the same patient.

This G repeat sequence is targeted by the highly conserved RepG sRNA (Regulator of polymeric G repeats) (Pernitzsch et al., 2014). The span of the G repeat determines the interaction of *tlpB* mRNA with RepG. Regulation is at the level of translation and is a fine-tuning system rather than being a binary on/off decision. Base-pairing of RepG to *tlpB* mRNA occurs in the 5′UTR, upstream of the RBS. Thus, translational attenuation is probably conferred by structural rearrangements and/or occlusion of ribosome stand-by sites. In addition, RepG is reported to diminish *tlpB* mRNA stability, indicating that the dimerization event enhances degradation. In addition to *tlpB*, RepG likely has a larger targetome, with which it probably interacts via its C/U laden terminator loop.

### 5′*ureB*-Regulatory RNA

Copious production of urease is a key mechanism that enables *H. pylori* to survive in the acidic gastric environment (Mobley, 1996; Graham and Miftahussurur, 2018). Urease metabolizes the available urea to ammonia and bicarbonate, both of which serve as buffer to maintain a healthy pH in the bacterial cell. Expressed from the same operon, UreA and UreB are the two subunits of the precursor urease apoenzyme. At the transcription level, *ureAB* is positively regulated by the acid-activated TCS ArsRS, ensuring plentiful synthesis (constituting ~8% of total cell protein) at low pH (Pflock et al., 2005).

Additionally, restricted urease synthesis is also required at neutral or high pH (Wen et al., 2013). This is facilitated by the 5′*ureB*-sRNA, an antisense RNA transcribed from the *5*′*ureB* non-coding strand.

While the phosphorylated (in acidic conditions) response regulator ArsR promotes sense *ureAB* transcription, the unphosphorylated (in neutral/alkaline conditions) protein upregulates the antisense 5′*ureB*-sRNA expression. It is observed that when the 5′*ureB*-sRNA is expressed (i.e., at neutral to high pH), the sense *ureAB* dicistronic mRNA is shortened to only 1400 nt instead of the regular 2700 nt transcript found at low pH, capable of synthesizing both subunits of the apoenzyme. Mechanistic explanation is that the antisense 5′*ureB*-sRNA base-pairs with the *ureAB* transcript, to promote transcription termination of the sense *ureAB* mRNA. A characteristic YUNR motif is essential for the initial annealing of the sense and antisense transcripts. Interestingly, this attenuation of transcription does not involve binding of Rho or even characteristic Rho-independent structures. As reported elsewhere, the asRNA binding leads to structural reassignments that ultimately destabilizes the RNA polymerase (Stork et al., 2007). At the same time, the termination of transcription is reported to be bona fide, rather than being caused by transcriptional interference of the sense and antisense transcripts being expressed at the same time (Shearwin et al., 2005). Finally, a low amount of the antisense 5′*ureB*-sRNA is enough to repress even high quantities of *ureAB* transcripts.

## Pseudomonas aeruginosa

Whenever it finds its host innate immunity weak, *P. aeruginosa* can cause opportunistic infections in virtually every human tissue (Lang et al., 2004; de Bentzmann and Plesiat, 2011). The most notable among these infection sites are the lungs of CF patients, where *P. aeruginosa* forms persistent biofilms (Hoiby et al., 2010; Smith et al., 2017). Once established, usually by late adolescence of the patient, these infections are almost impossible to eradicate and contribute largely to the decreased quality of life and life expectancy of these patients (Emerson et al., 2002; Bjarnsholt et al., 2009).

Multiple host environmental factors promote *P. aeruginosa* biofilm infections in the CF lungs. Among these is the viscous mucus, a hallmark of CF airway environment, which provides a favorable niche for microbial pathogens to thrive (Matsui et al., 2006; Moreau-Marquis et al., 2008). Additionally, the microorganisms induce a huge immune response by recruiting leucocytes to the area. The leucocytes however are failing to eliminate the infection and rather cause extensive tissue damage. This “frustrated phagocytosis” (Conese et al., 2003; Alexis et al., 2006; Simonin-Le Jeune et al., 2013; Okkotsu et al., 2014) further contributes to disease by providing extracellular DNA, aiding in the biofilm formation process (Tolker-Nielsen and Høiby, 2009; Fuxman Bass et al., 2010).

The other host-associated factors that will favor biofilm formation by *P. aeruginosa* include altered iron and oxygen availability in the CF airways (Moreau-Marquis et al., 2008). *P. aeruginosa* senses and responds adequately to such environmental cues. For example, motility and Type 3 Secretion System (T3SS), a machinery to produce and inject toxins directly into the host cytoplasm by a multiprotein syringe complex (Hauser, 2009), are downregulated while production of exopolysaccharides is increased (Furukawa et al., 2006; Folkesson et al., 2012; Winstanley et al., 2016). These behavioral changes are facilitated by modulation of gene expression, mainly carried out by sRNAs at the post-transcriptional level (Vakulskas et al., 2015). Therefore, it is not surprising that more than 570 sRNAs are reported to be expressed by *P. aeruginosa* (Pita et al., 2018).

Below, we explore some of the regulatory RNAs that play major roles in the switch from the planktonic (free swimming) acute lifestyle of *P. aeruginosa* to its biofilm lifestyle, characteristic of CF lung infections.

### Rsm Signaling

Almost a tenth of *P. aeruginosa* transcriptome is part of the Rsm regulon. This Rsm (regulator of secondary metabolites) regulon acts as a posttranscriptional regulatory system that controls multiple virulence determinants, ultimately governing the transition between the acutely toxic planktonic and the chronic biofilm growth modes (Vakulskas et al., 2015; Janssen et al., 2018).

The key components of the Rsm system are two RNA binding proteins, RsmA, and RsmF (also known as RsmN), which are orthologs to the *E. coli* CsrA (Carbon storage regulator A) protein. RsmA and RsmF share 31% identity and a conserved arginine is critical for their RNA binding activity (Janssen et al., 2018). Both proteins can directly bind target mRNAs and positively or negatively affect transcript stability and/or translation.

Presence of one (RsmA) and two (RsmF) conserved GGA motifs is important for target recognition (Romero et al., 2018). Both RsmA and RsmF activates the acute phenotype (T3SS, pilus, etc.) and represses the biofilm features (Pel and Psl exopolysaccharides, T6SS expression etc.).

These RNA binding proteins are tightly regulated by sRNAs RsmV, RsmW, RsmY, and RsmZ, also part of the Rsm system (Janssen et al., 2018). The sRNAs RsmV, RsmW, RsmY, and RsmZ can bind RsmA and RsmF by their GGA consensus sequence and sequester them away from their mRNA targets. Thus, these sRNAs are activators of the chronic biofilm-lifestyle phenotype while being repressors of the acute one. It is noteworthy that despite the apparent redundancy among the four similar acting sRNAs constituents of the Rsm system, variations do exist in their binding affinities. For example, RsmY and RsmZ have 10 times stronger affinity for RsmA than for RsmF. Moreover, their expression patterns will differ (Janssen et al., 2018).

Further, some differences have been observed in the regulons of RsmA vs. RsmF, despite their overall similar phenotypic regulatory patterns (Table 1) (Brencic and Lory, 2009; Romero et al., 2018). This highlights the critical need for fine-tuning mechanisms alongside major decision-making on-off switches, given the heterogeneity in the CF lung environment (Wei and Ma, 2013; Janssen et al., 2018).

Environmental regulation of the Rsm cascade, at least in part, is facilitated by the GacAS TCS (Brencic et al., 2009). The GacA response regulator, when phosphorylated by the sensor histidine kinase GacS, directly binds to the promoters of *rsmY* and *rsmZ* and activates their transcription. Two other membrane associated proteins LadS and RetS activate and inhibit GacA phosphorylation, respectively (Williams McMackin et al., 2019). LadS is stimulated by high calcium in the extracellular milieu (Broder et al., 2016). Further, recent work (Chakravarty et al., 2017) has reported that the inner membrane magnesium transporter MgtE, whose own expression is augmented during antibiotic pressure (Redelman et al., 2014) and low magnesium (both signals present in CF airway) (Coffey et al., 2014; Santi et al., 2016), signals through GacS to increase *rsmYZ* transcription. GacAS thus represents a hub of environmental regulation of the Rsm signaling. Contrary to RsmYZ, the sRNAs RsmW, and RsmV are not upregulated by GacAS (Janssen et al., 2018).

To add to the complex regulation of the Rsm regulon, another protein, the polynucleotide phosphorylase (PNPase) stabilizes both RsmY and RsmZ (Chen et al., 2016). Furthermore, *rsmYZ* transcription is inhibited by TspR, which acts through RetS (Williams McMackin et al., 2019). *rsmZ* transcription is also regulated by MvaT, and by BswR which thwarts the negative effects of MvaT (Williams McMackin et al., 2019). Another important sRNA implicated in T3SS repression is CrcZ, which sequesters both Crc and RsmF and activate T3SS gene expression (Sonnleitner et al., 2009; Williams McMackin et al., 2019).

### RNA Based Regulation of Quorum Sensing

#### Real

The conserved 100 nt sRNA ReaL (Regulator of alkyl quinolone) is involved in QS regulation in *P. aeruginosa* (Carloni et al., 2017). It is under the negative regulation of the Las QS system and activates translation of the *pqsC* transcript, thereby connecting the two QS systems important for modulating pleiotropic virulence phenotypes. ReaL is also under RpoS regulation and is therefore expressed strongly in stationary growth phase. Consistent with the phenotypes generally observed in isolates from CF lungs, ReaL downregulates swarming but increases biofilm formation and secretion of pyocyanin and pyoverdine (Meyer et al., 1996; Lau et al., 2004).

#### PhrS

The stationary phase of growth, characteristic of CF airway infections, sees another sRNA being upregulated: PhrS (Folsom et al., 2010; Sonnleitner et al., 2011). PhrS transcription is also activated through the oxygen responsive DNA binding ANR protein in low oxygen conditions, typical of the CF lungs (Zimmermann et al., 1991). Interestingly Hfq is required for steady state levels of PhrS, not because Hfq promotes its stability, but because it is required for ANR production. Consistent with its expression in CF lung-like conditions, it increases pyocyanin production. Finally, PhrS facilitates synthesis of the QS-related transcriptional regulator protein PqsR (Brouwer et al., 2014) by directly binding to it and activating translation of a short ORF element located upstream of the *pqsR* mRNA. This ORF is translationally joined with the *pqsR* transcript (Sonnleitner et al., 2011).

#### PrrF1 and PrrF2

A major sRNA regulatory system is encoded by the *prrF* locus in *P. aeruginosa* (Reinhart et al., 2017). The genes *prrF1* and *prrF2* are 95% identical in sequence and are located adjacent to each other in the genome, separated just by 95 bases (Djapgne et al., 2018). They are functional homologs of *E. coli* RyhB sRNA, and as such, both these sRNAs play key roles in iron homeostasis and in virulence (Nelson et al., 2019). PrrF sRNAs are transcriptionally upregulated under low iron conditions, during which they repress synthesis of non-essential iron-requiring proteins like SodB (Reinhart et al., 2015). Both PrrF1 (116 nt) and PrrF2 (114 nt) regulate QS in *P. aeruginosa* by base pairing with the *antR* mRNA and blocking its translation (Oglesby et al., 2008). AntR is a transcription factor that activates transcription of loci *antABC* and *catBCA*, responsible for breakdown of anthanilate, a precursor compound of the alkyl quinolone signaling molecule (Pita et al., 2018).

The apparent redundancy in structure and function of the two PrrF sRNAs can be rationalized by the finding that different signaling cascades, other than iron availability, may differentially regulate these two genes, thereby fine-tuning gene expression in response to slight variations in the extra and intracellular environment. For example, the AlgZR TCS, very important in rendering the CF lung mucoid phenotype in *P. aeruginosa* (Williams McMackin et al., 2019), activates the *prrF2* promoter but not the *prrF1* one (Little et al., 2018). Also, the tandem repeat structure of the *prrF* genes facilitate expression of another sRNA, PrrH, known to be involved in heme metabolism (Reinhart et al., 2015, 2017).

### ErsA

The envelope stress responsive sRNA A (ErsA) is activated by the envelope stress responsive factor Sigma22 (σ^22^) (AlgT/U) (Falcone et al., 2018). Additional CF lung signals like scarce iron and low oxygen both drives its transcription.

The first role of ErsA is direct base-pairing to the RBS of *algC* and repressing its translation in a Hfq-dependent manner. AlgC provides sugar residues for downstream synthesis of exopolysaccharides of the biofilm matrix (Okkotsu et al., 2014). Thus, through AlgC, ErsA is part of a feed forward cycle involved in biofilm polysaccharide formation.

The second role of ErsA is to directly bind the 5′UTR of the *oprD* mRNA and negatively regulate its translation (Li et al., 2012). Result of this regulation includes, but is not limited to, reducing influx of carbapenem antibiotics into the cell. This is important, given the widespread antibiotic resistance of CF airway-associated *P. aeruginosa*.

Interestingly, another sRNA called Sr0161 was also reported to repress *oprD* expression by the same mechanism (Zhang et al., 2017). Additionally, Sr0161 represses T3SS, consistent with its role in shaping *P. aeruginosa* phenotypes suited for the CF lung environment. The same study identified yet another sRNA, Sr006, which increases bacterial recalcitrance to polymyxin as well as decreases the pro-inflammatory profiles of the lipopolysaccharide, which can be considered important adaptations to the CF lung niche.

## Conclusion

Robust approaches for characterizing sRNA targetomes in bacteria have revolutionized our understanding of the gene regulatory patterns facilitated by these regulatory RNAs and their associated chaperones such as Hfq (Santiago-Frangos and Woodson, 2018). Development of techniques such as MAPS (Lalaouna et al., 2017), RilSeq (Melamed et al., 2018), ClipSeq (Andresen and Holmqvist, 2018), and Grad-seq (Smirnov et al., 2016) techniques are important milestones in the field. Additionally, obtaining information about sRNA-based regulation even at the single cell level is now possible, but nevertheless needs improvement (Saliba et al., 2014). We now have significant knowledge about RNA mediated regulation in a wide range of bacterial pathogens (Table 1, Figure 1) that has the potential to tremendously bolster the development of therapeutic approaches targeting these signaling pathways. This is critical, given the rapid expansion of antibiotic resistance in bacteria (Ventola, 2015; Hofer, 2019) and increasing ineffectiveness of existing antimicrobial treatments.

There are certain considerations when targeting sRNAs for developing antimicrobial therapeutics. First, sRNAs and their mechanisms of action are often not conserved (Richter and Backofen, 2012; Colameco and Elliot, 2017) and thus antibiotics targeting a certain sRNA might have limited spectrum. Secondly, lack of defined structural configurations in sRNAs like that of rRNAs and tRNAs (both of which are targets of numerous known antibiotics Chopra and Reader, 2014; Hong et al., 2014), makes the design of small molecule inhibitors challenging. In this regard, as certain studies (El-Mowafi et al., 2014) have already addressed this, sRNA chaperones such as Hfq might be a more lucrative target because of its conserved three-dimensional structure across bacterial species. Finally, most sRNAs act as fine tuning regulators of gene expression rather than as a binary on/off switch. This limits their promise in being a molecule target that can decisively clear an infection. Rather, targeting sRNAs could likely be a potential way to bolster conventional antibiotic strategies. Nevertheless, studying mechanisms of sRNA action, give us information on gene regulation right at the nucleotide resolution. This has been exploited in studies exploring “nucleotide-based antimicrobials” (Nikravesh et al., 2007).

The prospects of antimicrobial development demonstrate more potential with riboswitches. On the one hand, often riboswitches dictate major metabolic transitions (for example, see ethanolamine utilization by a riboswitch in *L. monocytogenes* described previously), rather than functioning only as a fine tuner of gene expression as sRNAs do. On the other hand, riboswitches, by nature, are excellent binders of small ligands. Another major advantage with riboswitches is that they have so far been never found in humans (Colameco and Elliot, 2017), and thus greatly reduce the chances of host toxicity. Though still in its infancy, there have been a few studies on targeting riboswitches for antimicrobial development. Some of them are summarized in Table 2. Such attempts should continue to grow and improve, as our knowledge about the occurrence and mode of action of more riboregulatory agents increases.

**Table 2:** Riboswitches explored as targets.

**Riboswitch**	**References**
FMN	Lee et al., 2009; Howe et al., 2015
*glmS*	Mayer and Famulok, 2006; Fei et al., 2014
Guanine-binding riboswitch	Kim et al., 2009; Mulhbacher et al., 2010
Cyclic di-GMP riboswitch	Furukawa et al., 2012
T-box riboswitch	Means et al., 2006; Anupam et al., 2008
Thiamine pyrophosphate riboswitch	Sudarsan et al., 2005
Lysine riboswitch	Sudarsan et al., 2003

## Author Contributions

All authors listed have made a substantial, direct and intellectual contribution to the work, and approved it for publication.

### Conflict of Interest

The authors declare that the research was conducted in the absence of any commercial or financial relationships that could be construed as a potential conflict of interest.

## References

[B1] Alexis N. E. Muhlebach M. S. Peden D. B. Noah T. L. (2006). Attenuation of host defense function of lung phagocytes in young cystic fibrosis patients. J. Cyst. Fibros. 5, 17–25. 10.1016/j.jcf.2005.11.001 16356787 PMC1764441

[B2] Anderson G. G. Palermo J. J. Schilling J. D. Roth R. Heuser J. Hultgren S. J. (2003). Intracellular bacterial biofilm-like pods in urinary tract infections. Science 301, 105–107. 10.1126/science.1084550 12843396

[B3] Andresen L. Holmqvist E. (2018). CLIP-Seq in bacteria: global recognition patterns of bacterial RNA-binding proteins. Meth. Enzymol. 612, 127–145. 10.1016/bs.mie.2018.08.008 30502939

[B4] Antunes L. C. Ferreira R. B. Buckner M. M. Finlay B. B. (2010). Quorum sensing in bacterial virulence. Microbiology 156(Pt 8), 2271–2282. 10.1099/mic.0.038794-0 20488878

[B5] Anupam R. Nayek A. Green N. J. Grundy F. J. Henkin T. M. Means J. A.. (2008). 4,5-Disubstituted oxazolidinones: high affinity molecular effectors of RNA function. Bioorg. Med. Chem. Lett. 18, 3541–3544. 10.1016/j.bmcl.2008.05.015 18502126 PMC2526248

[B6] Archambaud C. Nahori M.-A. Soubigou G. Bécavin C. Laval L. Lechat P.. (2012). Impact of lactobacilli on orally acquired listeriosis. Proc. Natl. Acad. Sci. U.S.A. 109, 16684–16689. 10.1073/pnas.1212809109 23012479 PMC3478606

[B7] Arnion H. Korkut D. N. Masachis Gelo S. Chabas S. Reignier J. Iost I.. (2017). Mechanistic insights into type I toxin antitoxin systems in *Helicobacter pylori*: the importance of mRNA folding in controlling toxin expression. Nucleic Acids Res. 45, 4782–4795. 10.1093/nar/gkw1343 28077560 PMC5416894

[B8] Bakaletz L. O. (2007). Bacterial biofilms in otitis media: evidence and relevance. J. Pediatric Infect. Dis. Soc. 26, S17–19. 10.1097/INF.0b013e318154b273 18049376

[B9] Banerjee G. Ray A. K. (2016). The talking language in some major Gram-negative bacteria. Arch. Microbiol. 198, 489–499. 10.1007/s00203-016-1220-x 27062655

[B10] Banerjee G. Ray A. K. (2017). Quorum-sensing network-associated gene regulation in Gram-positive bacteria. Acta Microbiol. Immunol. Hung. 64, 439–453. 10.1556/030.64.2017.040 29243493

[B11] Becker R. E. Bubeck Wardenburg J. (2015). *Staphylococcus aureus* and the skin: a longstanding and complex interaction. Skinmed 13, 111-119; quiz 120. 26137737

[B12] Begun J. Gaiani J. M. Rohde H. Mack D. Calderwood S. B. Ausubel F. M.. (2007). Staphylococcal biofilm exopolysaccharide protects against *Caenorhabditis elegans* immune defenses. PLoS Pathog. 3:e57. 10.1371/journal.ppat.0030057 17447841 PMC1853117

[B13] Bjarnsholt T. Jensen P. O. Fiandaca M. J. Pedersen J. Hansen C. R. Andersen C. B.. (2009). *Pseudomonas aeruginosa* biofilms in the respiratory tract of cystic fibrosis patients. Pediatr. Pulmonol. 44, 547–558. 10.1002/ppul.21011 19418571

[B14] Boisset S. Geissmann T. Huntzinger E. Fechter P. Bendridi N. Possedko M.. (2007). *Staphylococcus aureus* RNAIII coordinately represses the synthesis of virulence factors and the transcription regulator Rot by an antisense mechanism. Genes Dev. 21, 1353–1366. 10.1101/gad.423507 17545468 PMC1877748

[B15] Brencic A. Lory S. (2009). Determination of the regulon and identification of novel mRNA targets of *Pseudomonas aeruginosa* RsmA. Mol. Microbiol. 72, 612–632. 10.1111/j.1365-2958.2009.06670.x 19426209 PMC5567987

[B16] Brencic A. McFarland K. A. McManus H. R. Castang S. Mogno I. Dove S. L.. (2009). The GacS/GacA signal transduction system of *Pseudomonas aeruginosa* acts exclusively through its control over the transcription of the RsmY and RsmZ regulatory small RNAs. Mol. Microbiol. 73, 434–445. 10.1111/j.1365-2958.2009.06782.x 19602144 PMC2761719

[B17] Broder U. N. Jaeger T. Jenal U. (2016). LadS is a calcium-responsive kinase that induces acute-to-chronic virulence switch in *Pseudomonas aeruginosa*. Nat. Microbiol. 2:16184. 10.1038/nmicrobiol.2016.184 27775685

[B18] Bronesky D. Wu Z. Marzi S. Walter P. Geissmann T. Moreau K.. (2016). *Staphylococcus aureus* RNAIII and its regulon link quorum sensing, stress responses, metabolic adaptation, and regulation of virulence gene expression. Annu. Rev. Microbiol. 70, 299–316. 10.1146/annurev-micro-102215-095708 27482744

[B19] Brouwer S. Pustelny C. Ritter C. Klinkert B. Narberhaus F. Haussler S. (2014). The PqsR and RhlR transcriptional regulators determine the level of *Pseudomonas* quinolone signal synthesis in *Pseudomonas aeruginosa* by producing two different *pqsABCDE* mRNA isoforms. J. Bacteriol. 196, 4163–4171. 10.1128/JB.02000-14 25225275 PMC4248879

[B20] Calo L. Passali G. C. Galli J. Fadda G. Paludetti G. (2011). Role of biofilms in chronic inflammatory diseases of the upper airways. Adv. Otorhinolaryngol. 72, 93–96. 10.1159/000324622 21865700

[B21] Carloni S. Macchi R. Sattin S. Ferrara S. Bertoni G. (2017). The small RNA ReaL: a novel regulatory element embedded in the *Pseudomonas aeruginosa* quorum sensing networks. Environ. Microbiol. 19, 4220–4237. 10.1111/1462-2920.13886 28799693

[B22] Chabelskaya S. Gaillot O. Felden B. (2010). A *Staphylococcus aureus* small RNA Is required for bacterial virulence and regulates the expression of an immune-evasion molecule. PLoS Pathog. 6:e1000927. 10.1371/journal.ppat.1000927 20532214 PMC2880579

[B23] Chakravarty S. Melton C. N. Bailin A. Yahr T. L. Anderson G. G. (2017). *Pseudomonas aeruginosa* magnesium transporter MgtE inhibits type III secretion system gene expression by stimulating *rsmYZ* transcription. J. Bacteriol. 199:e00268-17. 10.1128/JB.00268-17 28847924 PMC5686585

[B24] Charlebois A. Jacques M. Archambault M. (2016). Comparative transcriptomic analysis of *Clostridium perfringens* biofilms and planktonic cells. Avian Pathol. 45, 593–601. 10.1080/03079457.2016.1189512 27207477

[B25] Chavez-Dozal A. Gorman C. Nishiguchi M. K. (2015). Proteomic and metabolomic profiles demonstrate variation among free-living and symbiotic *Vibrio fischeri* biofilms. BMC Microbiol. 15:226. 10.1186/s12866-015-0560-z 26494154 PMC4619220

[B26] Chen L. Wen Y. M. (2011). The role of bacterial biofilm in persistent infections and control strategies. Int. J. Oral Sci. 3, 66–73. 10.4248/IJOS11022 21485310 PMC3469879

[B27] Chen R. Weng Y. Zhu F. Jin Y. Liu C. Pan X.. (2016). Polynucleotide Phosphorylase regulates multiple virulence factors and the stabilities of small RNAs RsmY/Z in *Pseudomonas aeruginosa*. Front. Microbiol. 7:247. 10.3389/fmicb.2016.00247 26973625 PMC4773659

[B28] Chopra S. Reader J. (2014). tRNAs as antibiotic targets. Int. J. Mol. Sci. 16, 321–349. 10.3390/ijms16010321 25547494 PMC4307249

[B29] Christiansen J. K. Nielsen J. S. Ebersbach T. Valentin-Hansen P. Sogaard-Andersen L. Kallipolitis B. H. (2006). Identification of small Hfq-binding RNAs in *Listeria monocytogenes*. RNA. 12, 1383–1396. 10.1261/rna.49706 16682563 PMC1484441

[B30] Coffey B. M. Akhand S. S. Anderson G. G. (2014). MgtE is a dual-function protein in *Pseudomonas aeruginosa*. Microbiology. 160(Pt 6), 1200–1213. 10.1099/mic.0.075275-0 24722909

[B31] Colameco S. Elliot M. A. (2017). Non-coding RNAs as antibiotic targets. Biochem. Pharmacol. 133, 29–42. 10.1016/j.bcp.2016.12.015 28012959

[B32] Conese M. Copreni E. Gioia S. D. Rinaldis P. D. Fumarulo R. (2003). Neutrophil recruitment and airway epithelial cell involvement in chronic cystic fibrosis lung disease. J. Cyst. Fibros. 2, 129–135. 10.1016/S1569-1993(03)00063-8 15463861

[B33] Corey G. R. (2009). *Staphylococcus aureus* bloodstream infections: definitions and treatment. Clin. Infect. Dis. 48 (Suppl. 4), S254–259. 10.1086/598186 19374581

[B34] Cossart P. (2011). Illuminating the landscape of host-pathogen interactions with the bacterium *Listeria monocytogenes*. Proc. Natl. Acad. Sci. U.S.A. 108, 19484–19491. 10.1073/pnas.1112371108 22114192 PMC3241796

[B35] Costerton J. W. Lewandowski Z. Caldwell D. E. Korber D. R. Lappin-Scott H. M. (1995). Microbial biofilms. Annu. Rev. Microbiol. 49, 711–745. 10.1146/annurev.mi.49.100195.003431 8561477

[B36] Croxen M. A. Sisson G. Melano R. Hoffman P. S. (2006). The *Helicobacter pylori* chemotaxis receptor TlpB (HP0103) is required for pH taxis and for colonization of the gastric mucosa. J. Bacteriol. 188, 2656–2665. 10.1128/JB.188.7.2656-2665.2006 16547053 PMC1428400

[B37] Dayan G. H. Mohamed N. Scully I. L. Cooper D. Begier E. Eiden J.. (2016). *Staphylococcus aureus*: the current state of disease, pathophysiology and strategies for prevention. Expert Rev. Vaccines 15, 1373–1392. 10.1080/14760584.2016.1179583 27118628

[B38] de Bentzmann S. Plesiat P. (2011). The *Pseudomonas aeruginosa* opportunistic pathogen and human infections. Environ. Microbiol. 13, 1655–1665. 10.1111/j.1462-2920.2011.02469.x 21450006

[B39] de las Heras A. Cain R. J. Bielecka M. K. Vazquez-Boland J. A. (2011). Regulation of *Listeria* virulence: PrfA master and commander. Curr. Opin. Microbiol. 14, 118–127. 10.1016/j.mib.2011.01.005 21388862

[B40] Deng Z. Liu Z. Bi Y. Wang X. Zhou D. Yang R.. (2014). Rapid degradation of Hfq-free RyhB in *Yersinia pestis* by PNPase independent of putative ribonucleolytic complexes. BioMed Res. Int. 2014, 798918–798918. 10.1155/2014/798918 24818153 PMC4003864

[B41] Deng Z. Meng X. Su S. Liu Z. Ji X. Zhang Y.. (2012). Two sRNA RyhB homologs from *Yersinia pestis* biovar microtus expressed *in vivo* have differential Hfq-dependent stability. Res. Microbiol. 163, 413–418. 10.1016/j.resmic.2012.05.006 22659336

[B42] Djapgne L. Panja S. Brewer L. K. Gans J. H. Kane M. A. Woodson S. A.. (2018). The *Pseudomonas aeruginosa* PrrF1 and PrrF2 small regulatory RNAs promote 2-Alkyl-4-Quinolone production through redundant regulation of the *antR* mRNA. J. Bacteriol. 200:e00704-17. 10.1128/JB.00704-17 29507088 PMC5915787

[B43] do Vale A. Cabanes D. Sousa S. (2016). Bacterial toxins as pathogen weapons against phagocytes. Front. Microbiol. 7:42. 10.3389/fmicb.2016.00042 26870008 PMC4734073

[B44] Donlan R. M. (2008). Biofilms on central venous catheters: is eradication possible? Curr. Top. Microbiol. Immunol. 322, 133–161. 10.1007/978-3-540-75418-3_7 18453275

[B45] Dos Santos P. T. Menendez-Gil P. Sabharwal D. Christensen J. H. Brunhede M. Z. Lillebaek E. M. S.. (2018). The small regulatory RNAs LhrC1-5 contribute to the response of *Listeria monocytogenes* to heme toxicity. Front. Microbiol. 9:599. 10.3389/fmicb.2018.00599 29636750 PMC5880928

[B46] El-Mowafi S. A. Alumasa J. N. Ades S. E. Keiler K. C. (2014). Cell-based assay to identify inhibitors of the Hfq-sRNA regulatory pathway. Antimicrob. Agents. Chemother. 58, 5500–5509. 10.1128/AAC.03311-14 25001303 PMC4135888

[B47] Emerson J. Rosenfeld M. McNamara S. Ramsey B. Gibson R. L. (2002). *Pseudomonas aeruginosa* and other predictors of mortality and morbidity in young children with Cystic Fibrosis. Pediatr. Pulmonol. 34, 91–100. 10.1002/ppul.10127 12112774

[B48] Falcone M. Ferrara S. Rossi E. Johansen H. K. Molin S. Bertoni G. (2018). The small RNA ErsA of *Pseudomonas aeruginosa* contributes to biofilm development and motility through post-transcriptional modulation of AmrZ. Front. Microbiol. 9:238. 10.3389/fmicb.2018.00238 29497413 PMC5819304

[B49] Fang F. C. Frawley E. R. Tapscott T. Vázquez-Torres A. (2016). Bacterial stress responses during host infection. Cell Host Microbe 20, 133–143. 10.1016/j.chom.2016.07.009 27512901 PMC4985009

[B50] Favre L. Ortalo-Magne A. Pichereaux C. Gargaros A. Burlet-Schiltz O. Cotelle V.. (2018). Metabolome and proteome changes between biofilm and planktonic phenotypes of the marine bacterium *Pseudoalteromonas lipolytica* TC8. Biofouling 34, 132–148. 10.1080/08927014.2017.1413551 29319346

[B51] Fechter P. Caldelari I. Lioliou E. Romby P. (2014). Novel aspects of RNA regulation in *Staphylococcus aureus*. FEBS Lett. 588, 2523–2529. 10.1016/j.febslet.2014.05.037 24873876

[B52] Fei X. Holmes T. Diddle J. Hintz L. Delaney D. Stock A.. (2014). Phosphatase-inert glucosamine 6-phosphate mimics serve as actuators of the *glmS* riboswitch. ACS Chem. Biol. 9, 2875–2882. 10.1021/cb500458f 25254431 PMC4273988

[B53] Felden B. Vandenesch F. Bouloc P. Romby P. (2011). The *Staphylococcus aureus* RNome and its commitment to virulence. PLoS Pathog. 7:e1002006. 10.1371/journal.ppat.1002006 21423670 PMC3053349

[B54] Fernandez Guerrero M. L. Gonzalez Lopez J. J. Goyenechea A. Fraile J. de Gorgolas M. (2009). Endocarditis caused by *Staphylococcus aureus*: a reappraisal of the epidemiologic, clinical, and pathologic manifestations with analysis of factors determining outcome. Medicine 88, 1–22. 10.1097/MD.0b013e318194da65 19352296

[B55] Folkesson A. Jelsbak L. Yang L. Johansen H. K. Ciofu O. Hoiby N.. (2012). Adaptation of *Pseudomonas aeruginosa* to the Cystic Fibrosis airway: an evolutionary perspective. Nat. Rev. Microbiol. 10, 841–851. 10.1038/nrmicro2907 23147702

[B56] Folsom J. P. Richards L. Pitts B. Roe F. Ehrlich G. D. Parker A.. (2010). Physiology of *Pseudomonas aeruginosa* in biofilms as revealed by transcriptome analysis. BMC Microbiol. 10:294. 10.1186/1471-2180-10-294 21083928 PMC2998477

[B57] Fox K. A. Ramesh A. Stearns J. E. Bourgogne A. Reyes-Jara A. Winkler W. C.. (2009). Multiple posttranscriptional regulatory mechanisms partner to control ethanolamine utilization in *Enterococcus faecalis*. Proc. Natl. Acad. Sci. U.S.A. 106, 4435–4440. 10.1073/pnas.0812194106 19246383 PMC2647976

[B58] Freitag N. E. (2009). Complete transcriptional profile of an environmental pathogen. Future Microbiol. 4, 779–782. 10.2217/fmb.09.56 19722832

[B59] Fuchs T. M. Eisenreich W. Heesemann J. Goebel W. (2012). Metabolic adaptation of human pathogenic and related nonpathogenic bacteria to extra- and intracellular habitats. FEMS Microbiol. Rev. 36, 435–462. 10.1111/j.1574-6976.2011.00301.x 22092350

[B60] Furukawa K. Gu H. Sudarsan N. Hayakawa Y. Hyodo M. Breaker R. R. (2012). Identification of ligand analogues that control c-di-GMP riboswitches. ACS Chem. Biol. 7, 1436–1443. 10.1021/cb300138n 22646696 PMC4140405

[B61] Furukawa S. Kuchma S. L. O'Toole G. A. (2006). Keeping their options open: acute versus persistent infections. J. Bacteriol. 188, 1211–1217. 10.1128/JB.188.4.1211-1217.2006 16452401 PMC1367219

[B62] Fuxman Bass J. I. Russo D. M. Gabelloni M. L. Geffner J. R. Giordano M. Catalano M.. (2010). Extracellular DNA: a major proinflammatory component of *Pseudomonas aeruginosa* biofilms. J. Immunol. 184, 6386–6395. 10.4049/jimmunol.0901640 20421641

[B63] Gall-Mas L. Fabbri A. Namini M. R. J. Givskov M. Fiorentini C. Krejsgaard T. (2018). The bacterial toxin CNF1 induces activation and maturation of human monocyte-derived Dendritic Cells. Int. J. Mol. Sci. 19:1408. 10.3390/ijms19051408 29738516 PMC5983691

[B64] Garcia-del Portillo F. Calvo E. D'Orazio V. Pucciarelli M. G. (2011). Association of ActA to peptidoglycan revealed by cell wall proteomics of intracellular *Listeria monocytogenes*. J. Biol. Chem. 286, 34675–34689. 10.1074/jbc.M111.230441 21846725 PMC3186376

[B65] Garsin D. A. (2010). Ethanolamine utilization in bacterial pathogens: roles and regulation. Nat. Rev. Microbiol. 8, 290–295. 10.1038/nrmicro2334 20234377 PMC2950637

[B66] Geisinger E. Adhikari R. P. Jin R. Ross H. F. Novick R. P. (2006). Inhibition of *rot* translation by RNAIII, a key feature of *agr* function. Mol. Microbiol. 61, 1038–1048. 10.1111/j.1365-2958.2006.05292.x 16879652

[B67] Geissmann T. Marzi S. Romby P. (2009). The role of mRNA structure in translational control in bacteria. RNA Biol. 6, 153–160. 10.4161/rna.6.2.8047 19885993

[B68] Gellatly S. L. Hancock R. E. W. (2013). *Pseudomonas aeruginosa*: new insights into pathogenesis and host defenses. Pathog. Dis. 67, 159–173. 10.1111/2049-632X.12033 23620179

[B69] Goltermann L. Tolker-Nielsen T. (2017). Importance of the exopolysaccharide matrix in antimicrobial tolerance of *Pseudomonas aeruginosa* aggregates. Antimicrob. Agents Chemother. 61, e02696–e02616. 10.1128/AAC.02696-16 28137803 PMC5365683

[B70] Graham D. Y. Miftahussurur M. (2018). *Helicobacter pylori* urease for diagnosis of *Helicobacter pylori* infection: a mini review. J. Adv. Res. 13, 51–57. 10.1016/j.jare.2018.01.006 30094082 PMC6077137

[B71] Green E. R. Mecsas J. (2016). Bacterial secretion systems: an overview. Microbiol. Spectr. 4:VMBF-0012-2015. 10.1128/microbiolspec.VMBF-0012-2015 26999395 PMC4804464

[B72] Groisman E. A. Mouslim C. (2006). Sensing by bacterial regulatory systems in host and non-host environments. Nat. Rev. Microbiol. 4, 705–709. 10.1038/nrmicro1478 16894339

[B73] Guillet J. Hallier M. Felden B. (2013). Emerging functions for the *Staphylococcus aureus* RNome. PLoS Pathog. 9:e1003767. 10.1371/journal.ppat.1003767 24348246 PMC3861533

[B74] Hall C. W. Mah T.-F. (2017). Molecular mechanisms of biofilm-based antibiotic resistance and tolerance in pathogenic bacteria. FEMS Microbiol. Rev. 41, 276–301. 10.1093/femsre/fux010 28369412

[B75] Haupt K. Reuter M. van den Elsen J. Burman J. Hälbich S. Richter J.. (2008). The *Staphylococcus aureus* protein Sbi acts as a complement inhibitor and forms a tripartite complex with host complement factor H and C3b. PLoS Pathog. 4:e1000250. 10.1371/journal.ppat.1000250 19112495 PMC2602735

[B76] Hauser A. R. (2009). The type III secretion system of *Pseudomonas aeruginosa*: infection by injection. Nat. Rev. Microbiol. 7, 654–665. 10.1038/nrmicro2199 19680249 PMC2766515

[B77] Heidrich N. Bauriedl S. Barquist L. Li L. Schoen C. Vogel J. (2017). The primary transcriptome of *Neisseria meningitidis* and its interaction with the RNA chaperone Hfq. Nucleic Acids Res. 45, 6147–6167. 10.1093/nar/gkx168 28334889 PMC5449619

[B78] Hofer U. (2019). The cost of antimicrobial resistance. Nat. Rev. Microbiol. 17, 3–3. 10.1038/s41579-018-0125-x 30467331

[B79] Hoiby N. Ciofu O. Bjarnsholt T. (2010). *Pseudomonas aeruginosa* biofilms in Cystic Fibrosis. Future Microbiol. 5, 1663–1674. 10.2217/fmb.10.125 21133688

[B80] Hong W. Zeng J. Xie J. (2014). Antibiotic drugs targeting bacterial RNAs. Acta Pharm. Sin. B 4, 258–265. 10.1016/j.apsb.2014.06.012 26579393 PMC4629089

[B81] Howe J. A. Wang H. Fischmann T. O. Balibar C. J. Xiao L. Galgoci A. M.. (2015). Selective small-molecule inhibition of an RNA structural element. Nature 526, 672–677. 10.1038/nature15542 26416753

[B82] Ingavale S. van Wamel W. Luong T. T. Lee C. Y. Cheung A. L. (2005). Rat/MgrA, a regulator of autolysis, is a regulator of virulence genes in *Staphylococcus aureus*. Infect. Immun. 73, 1423–1431. 10.1128/IAI.73.3.1423-1431.2005 15731040 PMC1064946

[B83] James G. A. Swogger E. Wolcott R. Pulcini E. Secor P. Sestrich J.. (2008). Biofilms in chronic wounds. Wound Repair Regen. 16, 37–44. 10.1111/j.1524-475X.2007.00321.x 18086294

[B84] Janssen K. H. Diaz M. R. Gode C. J. Wolfgang M. C. Yahr T. L. (2018). RsmV, a small noncoding regulatory RNA in *Pseudomonas aeruginosa* that sequesters RsmA and RsmF from target mRNAs. J. Bacteriol. 200, e00277–e00218. 10.1128/JB.00277-18 29866805 PMC6060366

[B85] Jia K. Wang G. Liang L. Wang M. Wang H. Xu X. (2017). Preliminary transcriptome analysis of mature biofilm and planktonic cells of *Salmonella enteritidis* exposure to acid stress. Front. Microbiol. 8:1861. 10.3389/fmicb.2017.01861 29018430 PMC5622974

[B86] Joo H. S. Chatterjee S. S. Villaruz A. E. Dickey S. W. Tan V. Y. Chen Y.. (2016). Mechanism of gene regulation by a *Staphylococcus aureus* toxin. mBio 7:e01579-16. 10.1128/mBio.01579-16 27795396 PMC5080381

[B87] Kaper J. B. Nataro J. P. Mobley H. L. T. (2004). Pathogenic *Escherichia coli*. Nat. Rev. Microbiol. 2, 123–140. 10.1038/nrmicro818 15040260

[B88] Khandige S. Kronborg T. Uhlin B. E. Møller-Jensen J. (2015). sRNA-mediated regulation of P-fimbriae phase variation in Uropathogenic *Escherichia coli*. PLoS Pathog. 11:e1005109. 10.1371/journal.ppat.1005109 26291711 PMC4546395

[B89] Kim E. Y. Jakobson C. M. Tullman-Ercek D. (2014). Engineering transcriptional regulation to control Pdu microcompartment formation. PLoS ONE 9:e113814. 10.1371/journal.pone.0113814 25427074 PMC4245221

[B90] Kim J. N. Blount K. F. Puskarz I. Lim J. Link K. H. Breaker R. R. (2009). Design and antimicrobial action of purine analogues that bind Guanine riboswitches. ACS Chem. Biol. 4, 915–927. 10.1021/cb900146k 19739679 PMC4140397

[B91] Kulesus R. R. Diaz-Perez K. Slechta E. S. Eto D. S. Mulvey M. A. (2008). Impact of the RNA chaperone Hfq on the fitness and virulence potential of Uropathogenic *Escherichia coli*. Infect. Immun. 76, 3019–3026. 10.1128/IAI.00022-08 18458066 PMC2446724

[B92] Kusters J. G. van Vliet A. H. Kuipers E. J. (2006). Pathogenesis of *Helicobacter pylori* infection. Clin. Microbiol. Rev. 19, 449–490. 10.1128/CMR.00054-05 16847081 PMC1539101

[B93] Lalaouna D. Prevost K. Eyraud A. Masse E. (2017). Identification of unknown RNA partners using MAPS. Methods 117, 28–34. 10.1016/j.ymeth.2016.11.011 27876680

[B94] Lane M. C. Mobley H. L. (2007). Role of P-fimbrial-mediated adherence in pyelonephritis and persistence of Uropathogenic *Escherichia coli* (UPEC) in the mammalian kidney. Kidney Int. 72, 19–25. 10.1038/sj.ki.5002230 17396114

[B95] Lang A. B. Horn M. P. Imboden M. A. Zuercher A. W. (2004). Prophylaxis and therapy of *Pseudomonas aeruginosa* infection in Cystic Fibrosis and immunocompromised patients. Vaccine 22 (Suppl. 1), S44–48. 10.1016/j.vaccine.2004.08.016 15576201

[B96] Lau G. W. Ran H. Kong F. Hassett D. J. Mavrodi D. (2004). *Pseudomonas aeruginosa* Pyocyanin is critical for lung infection in mice. Infect. Immun. 72, 4275–4278. 10.1128/IAI.72.7.4275-4278.2004 15213173 PMC427412

[B97] Lebreton A. Cossart P. (2016). RNA and protein-mediated control of *Listeria monocytogenes* virulence gene expression. RNA Biol. 14, 460–470. 10.1080/15476286.2016.1189069 27217337 PMC5449094

[B98] Lee E. R. Blount K. F. Breaker R. R. (2009). Roseoflavin is a natural antibacterial compound that binds to FMN riboswitches and regulates gene expression. RNA Biol. 6, 187–194. 10.4161/rna.6.2.7727 19246992 PMC5340298

[B99] Lee P.-C. Rietsch A. (2015). Fueling type III secretion. Trends Microbiol. 23, 296–300. 10.1016/j.tim.2015.01.012 25701111 PMC4417389

[B100] Leid J. G. Willson C. J. Shirtliff M. E. Hassett D. J. Parsek M. R. Jeffers A. K. (2005). The exopolysaccharide alginate protects *Pseudomonas aeruginosa* biofilm bacteria from IFN-γ-mediated macrophage killing. J. Immunol. 175, 7512–7518. 10.4049/jimmunol.175.11.7512 16301659

[B101] Li H. Luo Y. F. Williams B. J. Blackwell T. S. Xie C. M. (2012). Structure and function of OprD protein in *Pseudomonas aeruginosa*: from antibiotic resistance to novel therapies. Int. J. Med. Microbiol. 302, 63–68. 10.1016/j.ijmm.2011.10.001 22226846 PMC3831278

[B102] Libânio D. Dinis-Ribeiro M. Pimentel-Nunes P. (2015). *Helicobacter pylori* and microRNAs: relation with innate immunity and progression of preneoplastic conditions. World J. Clin. Oncol. 6, 111–132. 10.5306/wjco.v6.i5.111 26468448 PMC4600186

[B103] Lister J. L. Horswill A. R. (2014). *Staphylococcus aureus* biofilms: recent developments in biofilm dispersal. Front. Cell. Infect. Microbiol. 4:178. 10.3389/fcimb.2014.00178 25566513 PMC4275032

[B104] Little A. S. Okkotsu Y. Reinhart A. A. Damron F. H. Barbier M. Barrett B.. (2018). *Pseudomonas aeruginosa* AlgR phosphorylation status differentially regulates pyocyanin and pyoverdine production. mBio 9, e02318–e02317. 10.1128/mBio.02318-17 29382736 PMC5790918

[B105] Liu Y. Wu N. Dong J. Gao Y. Zhang X. Mu C.. (2010). Hfq is a global regulator that controls the pathogenicity of *Staphylococcus aureus*. PLoS ONE 5:e13069. 10.1371/journal.pone.0013069 20927372 PMC2947504

[B106] Loh E. Dussurget O. Gripenland J. Vaitkevicius K. Tiensuu T. Mandin P.. (2009). A trans-acting riboswitch controls expression of the virulence regulator PrfA in *Listeria monocytogenes*. Cell 139, 770–779. 10.1016/j.cell.2009.08.046 19914169

[B107] Loh E. Righetti F. Eichner H. Twittenhoff C. Narberhaus F. (2018). RNA thermometers in bacterial pathogens. Microbiol. Spectr. 6:RWR-0012-2017. 10.1128/microbiolspec.RWR-0012-2017 29623874 PMC11633587

[B108] Lopez-Causape C. Rojo-Molinero E. Macia M. D. Oliver A. (2015). The problems of antibiotic resistance in Cystic Fibrosis and solutions. Expert Rev. Respir. Med. 9, 73–88. 10.1586/17476348.2015.995640 25541089

[B109] Luong T. T. Dunman P. M. Murphy E. Projan S. J. Lee C. Y. (2006). Transcription profiling of the *mgrA* regulon in *Staphylococcus aureus*. J. Bacteriol. 188, 1899–1910. 10.1128/JB.188.5.1899-1910.2006 16484201 PMC1426550

[B110] Mah T.-F. O'Toole G. A. (2001). Mechanisms of biofilm resistance to antimicrobial agents. Trends Microbiol. 9, 34–39. 10.1016/S0966-842X(00)01913-2 11166241

[B111] Mariscotti J. F. Quereda J. J. Pucciarelli M. G. (2012). Contribution of sortase A to the regulation of *Listeria monocytogenes* LPXTG surface proteins. Int. Microbiol. 15, 43–51. 10.2436/20.1501.01.157 22837151

[B112] Massé E. Gottesman S. (2002). A small RNA regulates the expression of genes involved in iron metabolism in *Escherichia coli*. Proc. Natl. Acad. Sci. U.S.A. 99, 4620–4625. 10.1073/pnas.032066599 11917098 PMC123697

[B113] Matos R. G. Casinhas J. Barria C. Dos Santos R. F. Silva I. J. Arraiano C. M. (2017). The role of ribonucleases and sRNAs in the virulence of foodborne pathogens. Front. Microbiol. 8:910. 10.3389/fmicb.2017.00910 28579982 PMC5437115

[B114] Matsui H. Wagner V. E. Hill D. B. Schwab U. E. Rogers T. D. Button B.. (2006). A physical linkage between Cystic Fibrosis airway surface dehydration and *Pseudomonas aeruginosa* biofilms. Proc. Natl. Acad. Sci. U.S.A. 103, 18131–18136. 10.1073/pnas.0606428103 17116883 PMC1838718

[B115] Mayer G. Famulok M. (2006). High-throughput-compatible assay for *glmS* riboswitch metabolite dependence. Chembiochem 7, 602–604. 10.1002/cbic.200500490 16485317

[B116] McCaig L. F. McDonald L. C. Mandal S. Jernigan D. B. (2006). *Staphylococcus aureus*-associated skin and soft tissue infections in ambulatory care. Emerging Infect. Dis. 12, 1715–1723. 10.3201/eid1211.060190 17283622 PMC3372331

[B117] Means J. Katz S. Nayek A. Anupam R. Hines J. V. Bergmeier S. C. (2006). Structure-activity studies of oxazolidinone analogs as RNA-binding agents. Bioorg. Med. Chem. Lett. 16, 3600–3604. 10.1016/j.bmcl.2006.03.068 16603349

[B118] Melamed S. Faigenbaum-Romm R. Peer A. Reiss N. Shechter O. Bar A.. (2018). Mapping the small RNA interactome in bacteria using RIL-seq. Nat. Protoc. 13, 1–33. 10.1038/nprot.2017.115 29215635

[B119] Mellin J. R. Koutero M. Dar D. Nahori M. A. Sorek R. Cossart P. (2014). Riboswitches. Sequestration of a two-component response regulator by a riboswitch-regulated noncoding RNA. Science 345, 940–943. 10.1126/science.1255083 25146292

[B120] Mellin J. R. Tiensuu T. Becavin C. Gouin E. Johansson J. Cossart P. (2013). A riboswitch-regulated antisense RNA in *Listeria monocytogenes*. Proc. Natl. Acad. Sci. U.S.A. 110, 13132–13137. 10.1073/pnas.1304795110 23878253 PMC3740843

[B121] Meyer J. M. Neely A. Stintzi A. Georges C. Holder I. A. (1996). Pyoverdin is essential for virulence of *Pseudomonas aeruginosa*. Infect. Immun. 64, 518–523. 8550201 10.1128/iai.64.2.518-523.1996 PMC173795

[B122] Miao E. A. Mao D. P. Yudkovsky N. Bonneau R. Lorang C. G. Warren S. E.. (2010). Innate immune detection of the type III secretion apparatus through the NLRC4 inflammasome. Proc. Natl. Acad. Sci. U.S.A. 107, 3076–3080. 10.1073/pnas.0913087107 20133635 PMC2840275

[B123] Miller M. B. Bassler B. L. (2001). Quorum sensing in bacteria. Annu. Rev. Microbiol. 55, 165–199. 10.1146/annurev.micro.55.1.165 11544353

[B124] Mobley H. L. (1996). The role of *Helicobacter pylori* urease in the pathogenesis of gastritis and peptic ulceration. Aliment. Pharmacol. Ther. 10 (Suppl. 1), 57–64. 10.1046/j.1365-2036.1996.22164006.x 8730260

[B125] Moreau-Marquis S. Bomberger J. M. Anderson G. G. Swiatecka-Urban A. Ye S. O'Toole G. A.. (2008). The DeltaF508-CFTR mutation results in increased biofilm formation by *Pseudomonas aeruginosa* by increasing iron availability. Am. J. Physiol. Lung Cell Mol. Physiol. 295, L25–37. 10.1152/ajplung.00391.2007 18359885 PMC2494796

[B126] Morfeldt E. Taylor D. von Gabain A. Arvidson S. (1995). Activation of alpha-toxin translation in *Staphylococcus aureus* by the trans-encoded antisense RNA, RNAIII. EMBO J. 14, 4569–4577. 10.1002/j.1460-2075.1995.tb00136.x 7556100 PMC394549

[B127] Mulhbacher J. Brouillette E. Allard M. Fortier L. C. Malouin F. Lafontaine D. A. (2010). Novel riboswitch ligand analogs as selective inhibitors of guanine-related metabolic pathways. PLoS Pathog. 6:e1000865. 10.1371/journal.ppat.1000865 20421948 PMC2858708

[B128] Murphy E. R. Payne S. M. (2007). RyhB, an iron-responsive small RNA molecule, regulates *Shigella dysenteriae* virulence. Infect. Immun. 75, 3470–3477. 10.1128/IAI.00112-07 17438026 PMC1932958

[B129] Nechooshtan G. Elgrably-Weiss M. Sheaffer A. Westhof E. Altuvia S. (2009). A pH-responsive riboregulator. Genes Dev. 23, 2650–2662. 10.1101/gad.552209 19933154 PMC2779765

[B130] Nelson C. E. Huang W. Brewer L. K. Nguyen A. T. Kane M. A. Wilks A.. (2019). Proteomic Analysis of the *Pseudomonas aeruginosa* iron starvation response reveals PrrF small regulatory RNA-dependent iron regulation of twitching motility, amino acid metabolism, and zinc homeostasis proteins. J. Bacteriol. 201:e00754-18. 10.1128/JB.00754-18 30962354 PMC6531625

[B131] Nigaud Y. Cosette P. Collet A. Song P. C. Vaudry D. Vaudry H.. (2010). Biofilm-induced modifications in the proteome of *Pseudomonas aeruginosa* planktonic cells. Biochim. Biophys. Acta 1804, 957–966. 10.1016/j.bbapap.2010.01.008 20080211

[B132] Nikravesh A. Dryselius R. Faridani O. R. Goh S. Sadeghizadeh M. Behmanesh M.. (2007). Antisense PNA accumulates in *Escherichia coli* and mediates a long post-antibiotic effect. Mol. Ther. Nucleic Acids 15, 1537–1542. 10.1038/sj.mt.6300209 17534267

[B133] Noto M. J. Kreiswirth B. N. Monk A. B. Archer G. L. (2008). Gene acquisition at the insertion site for SCCmec, the genomic island conferring methicillin resistance in *Staphylococcus aureus*. J. Bacteriol. 190, 1276–1283. 10.1128/JB.01128-07 18083809 PMC2238224

[B134] Oglesby A. G. Farrow J. M. 3rd Lee J. H. Tomaras A. P. Greenberg E. P. Pesci E. C.. (2008). The influence of iron on *Pseudomonas aeruginosa* physiology: a regulatory link between iron and quorum sensing. J. Biol. Chem. 283, 15558–15567. 10.1074/jbc.M707840200 18424436 PMC2414296

[B135] Oglesby-Sherrouse A. G. Murphy E. R. (2013). Iron-responsive bacterial small RNAs: variations on a theme. Metallomics 5, 276–286. 10.1039/c3mt20224k 23340911 PMC3612141

[B136] Okkotsu Y. Little A. S. Schurr M. J. (2014). The Pseudomonas aeruginosa AlgZR two-component system coordinates multiple phenotypes. Front. Cell.Infect. Microbiol. 4:82. 10.3389/fcimb.2014.00082 24999454 PMC4064291

[B137] Olson M. E. Horswill A. R. (2013). *Staphylococcus aureus* osteomyelitis: bad to the bone. Cell Host Microbe 13, 629–631. 10.1016/j.chom.2013.05.015 23768487 PMC3732834

[B138] Omar A. Wright J. B. Schultz G. Burrell R. Nadworny P. (2017). Microbial biofilms and chronic wounds. Microorganisms 5:9. 10.3390/microorganisms5010009 28272369 PMC5374386

[B139] Oosthuizen M. C. Steyn B. Theron J. Cosette P. Lindsay D. von Holy A.. (2002). Proteomic analysis reveals differential protein expression by *Bacillus cereus* during biofilm formation. Appl. Environ. Microbiol. 68, 2770–2780. 10.1128/AEM.68.6.2770-2780.2002 12039732 PMC123966

[B140] Pannekoek Y. Huis In 't Veld R. A. Schipper K. Bovenkerk S. Kramer G. Brouwer M. C.. (2017). *Neisseria meningitidis* uses sibling small regulatory RNAs to switch from cataplerotic to anaplerotic metabolism. mBio 8:e02293-16. 10.1128/mBio.02293-16 28325760 PMC5362039

[B141] Papenfort K. Vogel J. (2014). Small RNA functions in carbon metabolism and virulence of enteric pathogens. Front. Cell. Infect. Microbiol. 4, 91. 10.3389/fcimb.2014.00091 25077072 PMC4098024

[B142] Parker D. Prince A. (2012). Immunopathogenesis of *Staphylococcus aureus* pulmonary infection. Semin. Immunopathol. 34, 281–297. 10.1007/s00281-011-0291-7 22037948 PMC3577067

[B143] Pernitzsch S. R. Sharma C. M. (2012). Transcriptome complexity and riboregulation in the human pathogen *Helicobacter pylori*. Front. Cell. Infect. Microbiol. 2:14. 10.3389/fcimb.2012.00014 22919606 PMC3417511

[B144] Pernitzsch S. R. Tirier S. M. Beier D. Sharma C. M. (2014). A variable homopolymeric G-repeat defines small RNA-mediated posttranscriptional regulation of a chemotaxis receptor in *Helicobacter pylori*. Proc. Natl. Acad. Sci. U.S.A. 111, E501–510. 10.1073/pnas.1315152111 24474799 PMC3910625

[B145] Pettersson J. Nordfelth R. Dubinina E. Bergman T. Gustafsson M. Magnusson K. E.. (1996). Modulation of virulence factor expression by pathogen target cell contact. Science 273, 1231–1233. 10.1126/science.273.5279.1231 8703058

[B146] Pflock M. Kennard S. Delany I. Scarlato V. Beier D. (2005). Acid-induced activation of the urease promoters is mediated directly by the ArsRS two-component system of *Helicobacter pylori*. Infect. Immun. 73, 6437–6445. 10.1128/IAI.73.10.6437-6445.2005 16177315 PMC1230922

[B147] Pita T. Feliciano J. R. Leitão J. H. (2018). Small noncoding regulatory RNAs from *Pseudomonas aeruginosa* and *Burkholderia cepacia* complex. Int. J. Mol. Sci. 19:3759. 10.3390/ijms19123759 30486355 PMC6321483

[B148] Pizarro-Cerda J. Kuhbacher A. Cossart P. (2012). Entry of *Listeria monocytogenes* in mammalian epithelial cells: an updated view. Cold Spring Harb. Perspect. Med. 2:a010009. 10.1101/cshperspect.a010009 23125201 PMC3543101

[B149] Porcheron G. Habib R. Houle S. Caza M. Lépine F. Daigle F.. (2014). The small RNA RyhB contributes to siderophore production and virulence of Uropathogenic *Escherichia coli*. Infect. Immun. 82, 5056–5068. 10.1128/IAI.02287-14 25245805 PMC4249264

[B150] Powers M. E. Bubeck Wardenburg J. (2014). Igniting the fire: *Staphylococcus aureus* virulence factors in the pathogenesis of sepsis. PLoS Pathog. 10:e1003871. 10.1371/journal.ppat.1003871 24550724 PMC3923759

[B151] Pucciarelli M. G. Calvo E. Sabet C. Bierne H. Cossart P. García-del Portillo F. (2005). Identification of substrates of the *Listeria monocytogenes* sortases A and B by a non-gel proteomic analysis. Proteomics 5, 4808–4817. 10.1002/pmic.200402075 16247833

[B152] Qin L. McCausland J. W. Cheung G. Y. C. Otto M. (2016). PSM-Mec-A virulence determinant that connects transcriptional regulation, virulence, and antibiotic resistance in Staphylococci. Front. Microbiol. 7:1293. 10.3389/fmicb.2016.01293 27597849 PMC4992726

[B153] Quereda J. J. Ortega Á. D. Pucciarelli M. G. García-del Portillo F. (2014). The *Listeria* small RNA Rli27 regulates a cell wall protein inside eukaryotic cells by targeting a long 5′-UTR variant. PLoS Genet. 10:e1004765. 10.1371/journal.pgen.1004765 25356775 PMC4214639

[B154] Radoshevich L. Cossart P. (2018). *Listeria monocytogenes*: towards a complete picture of its physiology and pathogenesis. Nat. Rev. Microbiol. 16, 32–46. 10.1038/nrmicro.2017.126 29176582

[B155] Redelman C. V. Chakravarty S. Anderson G. G. (2014). Antibiotic treatment of *Pseudomonas aeruginosa* biofilms stimulates expression of the magnesium transporter gene *mgtE*. Microbiology. 160(Pt 1), 165–178. 10.1099/mic.0.070144-0 24162608

[B156] Reinhart A. A. Nguyen A. T. Brewer L. K. Bevere J. Jones J. W. Kane M. A.. (2017). The *Pseudomonas aeruginosa* PrrF small RNAs regulate iron homeostasis during acute murine lung infection. Infect. Immun. 85, e00764–e00716. 10.1128/IAI.00764-16 28289146 PMC5400841

[B157] Reinhart A. A. Powell D. A. Nguyen A. T. O'Neill M. Djapgne L. Wilks A.. (2015). The *prrF*-encoded small regulatory RNAs are required for iron homeostasis and virulence of *Pseudomonas aeruginosa*. Infect. Immun. 83, 863–875. 10.1128/IAI.02707-14 25510881 PMC4333466

[B158] Richard A. L. Withey J. H. Beyhan S. Yildiz F. DiRita V. J. (2010). The *Vibrio cholerae* virulence regulatory cascade controls glucose uptake through activation of TarA, a small regulatory RNA. Mol. Microbiol. 78, 1171–1181. 10.1111/j.1365-2958.2010.07397.x 21091503 PMC3064952

[B159] Richter A. S. Backofen R. (2012). Accessibility and conservation: general features of bacterial small RNA-mRNA interactions? RNA Biol. 9, 954–965. 10.4161/rna.20294 22767260 PMC3495738

[B160] Romby P. Vandenesch F. Wagner E. G. (2006). The role of RNAs in the regulation of virulence-gene expression. Curr. Opin. Microbiol. 9, 229–236. 10.1016/j.mib.2006.02.005 16529986

[B161] Romero M. Silistre H. Lovelock L. Wright V. J. Chan K.-G. Hong K.-W.. (2018). Genome-wide mapping of the RNA targets of the *Pseudomonas aeruginosa* riboregulatory protein RsmN. Nucleic Acids Res. 46, 6823–6840. 10.1093/nar/gky324 29718466 PMC6061880

[B162] Romilly C. Lays C. Tomasini A. Caldelari I. Benito Y. Hammann P.. (2014). A non-coding RNA promotes bacterial persistence and decreases virulence by regulating a regulator in *Staphylococcus aureus*. PLoS Pathog. 10:e1003979. 10.1371/journal.ppat.1003979 24651379 PMC3961350

[B163] Ross J. A. Thorsing M. Lillebaek E. M. S. Teixeira Dos Santos P. Kallipolitis B. H. (2019). The LhrC sRNAs control expression of T cell-stimulating antigen TcsA in *Listeria monocytogenes* by decreasing *tcsA* mRNA stability. RNA Biol. 16, 270–281. 10.1080/15476286.2019.1572423 30706751 PMC6380316

[B164] Rutherford S. T. Bassler B. L. (2012). Bacterial quorum sensing: its role in virulence and possibilities for its control. Cold Spring Harb. Perspect. Med. 2:a012427. 10.1101/cshperspect.a012427 23125205 PMC3543102

[B165] Saliba A. E. Westermann A. J. Gorski S. A. Vogel J. (2014). Single-cell RNA-seq: advances and future challenges. Nucleic Acids Res. 42, 8845–8860. 10.1093/nar/gku555 25053837 PMC4132710

[B166] Santajit S. Indrawattana N. (2016). Mechanisms of antimicrobial resistance in ESKAPE pathogens. BioMed Res. Int. 2016:2475067. 10.1155/2016/2475067 27274985 PMC4871955

[B167] Santi M. Milani G. P. Simonetti G. D. Fossali E. F. Bianchetti M. G. Lava S. A. (2016). Magnesium in Cystic Fibrosis–systematic review of the literature. Pediatr. Pulmonol. 51, 196–202. 10.1002/ppul.23356 26663706

[B168] Santiago-Frangos A. Woodson S. A. (2018). Hfq chaperone brings speed dating to bacterial sRNA. Wiley Interdiscip. Rev. RNA 9:e1475. 10.1002/wrna.1475 29633565 PMC6002925

[B169] Schultze T. Hilker R. Mannala G. K. Gentil K. Weigel M. Farmani N.. (2015). A detailed view of the intracellular transcriptome of *Listeria monocytogenes* in murine macrophages using RNA-seq. Front. Microbiol. 6:1199. 10.3389/fmicb.2015.01199 26579105 PMC4627465

[B170] Sesto N. Wurtzel O. Archambaud C. Sorek R. Cossart P. (2012). The excludon: a new concept in bacterial antisense RNA-mediated gene regulation. Nat. Rev. Microbiol. 11, 75–82. 10.1038/nrmicro2934 23268228

[B171] Shearwin K. E. Callen B. P. Egan J. B. (2005). Transcriptional interference–a crash course. Trends Genet. 21, 339–345. 10.1016/j.tig.2005.04.009 15922833 PMC2941638

[B172] Sievers S. Lund A. Menendez-Gil P. Nielsen A. Storm Mollerup M. Lambert Nielsen S.. (2015). The multicopy sRNA LhrC controls expression of the oligopeptide-binding protein OppA in *Listeria monocytogenes*. RNA Biol. 12, 985–997. 10.1080/15476286.2015.1071011 26176322 PMC4615310

[B173] Sievers S. Sternkopf Lillebaek E. M. Jacobsen K. Lund A. Mollerup M. S. Nielsen P. K.. (2014). A multicopy sRNA of *Listeria monocytogenes* regulates expression of the virulence adhesin LapB. Nucleic Acids Res. 42, 9383–9398. 10.1093/nar/gku630 25034691 PMC4132741

[B174] Simonin-Le Jeune K. Le Jeune A. Jouneau S. Belleguic C. Roux P.-F. Jaguin M.. (2013). Impaired functions of macrophage from Cystic Fibrosis patients: CD11b, TLR-5 decrease and sCD14, inflammatory cytokines increase. PLoS ONE 8:e75667. 10.1371/journal.pone.0075667 24098711 PMC3787056

[B175] Singh S. Singh S. K. Chowdhury I. Singh R. (2017). Understanding the mechanism of bacterial biofilms resistance to antimicrobial agents. Open Microbiol. J. 11, 53–62. 10.2174/1874285801711010053 28553416 PMC5427689

[B176] Smirnov A. Förstner K. U. Holmqvist E. Otto A. Günster R. Becher D.. (2016). Grad-seq guides the discovery of ProQ as a major small RNA-binding protein. Proc. Natl. Acad. Sci. U.S.A. 113, 11591–11596. 10.1073/pnas.1609981113 27671629 PMC5068311

[B177] Smith W. D. Bardin E. Cameron L. Edmondson C. L. Farrant K. V. Martin I.. (2017). Current and future therapies for *Pseudomonas aeruginosa* infection in patients with Cystic Fibrosis. FEMS Microbiol. Lett. 364:fnx121. 10.1093/femsle/fnx121 28854668

[B178] Sonnleitner E. Abdou L. Haas D. (2009). Small RNA as global regulator of carbon catabolite repression in *Pseudomonas aeruginosa*. Proc. Natl. Acad. Sci. U.S.A. 106, 21866–21871. 10.1073/pnas.0910308106 20080802 PMC2799872

[B179] Sonnleitner E. Gonzalez N. Sorger-Domenigg T. Heeb S. Richter A. S. Backofen R.. (2011). The small RNA PhrS stimulates synthesis of the *Pseudomonas aeruginosa* quinolone signal. Mol. Microbiol. 80, 868–885. 10.1111/j.1365-2958.2011.07620.x 21375594

[B180] Stewart P. S. (2002). Mechanisms of antibiotic resistance in bacterial biofilms. Int. J. Med. Microbiol. 292, 107–113. 10.1078/1438-4221-00196 12195733

[B181] Stewart P. S. (2003). Diffusion in biofilms. J. Bacteriol. 185, 1485–1491. 10.1128/JB.185.5.1485-1491.2003 12591863 PMC148055

[B182] Stork M. Di Lorenzo M. Welch T. J. Crosa J. H. (2007). Transcription termination within the iron transport-biosynthesis operon of *Vibrio anguillarum* requires an antisense RNA. J. Bacteriol. 189, 3479–3488. 10.1128/JB.00619-06 17337574 PMC1855896

[B183] Sudarsan N. Cohen-Chalamish S. Nakamura S. Emilsson G. M. Breaker R. R. (2005). Thiamine pyrophosphate riboswitches are targets for the antimicrobial compound pyrithiamine. Chem. Biol. 12, 1325–1335. 10.1016/j.chembiol.2005.10.007 16356850

[B184] Sudarsan N. Wickiser J. K. Nakamura S. Ebert M. S. Breaker R. R. (2003). An mRNA structure in bacteria that controls gene expression by binding lysine. Genes Dev. 17, 2688–2697. 10.1101/gad.1140003 14597663 PMC280618

[B185] Sutherland I. W. (2001). Exopolysaccharides in biofilms, flocs and related structures. Water Sci. Technol. 43, 77–86. 10.2166/wst.2001.0345 11381975

[B186] Svensson S. L. Sharma C. M. (2016). Small RNAs in bacterial virulence and communication. Microbiol. Spectr. 4:VMBF-0028-2015. 10.1128/microbiolspec.VMBF-0028-2015 27337442

[B187] Terlizzi M. E. Gribaudo G. Maffei M. E. (2017). UroPathogenic *Escherichia coli* (UPEC) infections: virulence factors, bladder responses, antibiotic, and non-antibiotic antimicrobial strategies. Front. Microbiol. 8:1566. 10.3389/fmicb.2017.01566 28861072 PMC5559502

[B188] Testerman T. L. Morris J. (2014). Beyond the stomach: an updated view of *Helicobacter pylori* pathogenesis, diagnosis, and treatment. World J. Gastroenterol. 20, 12781–12808. 10.3748/wjg.v20.i36.12781 25278678 PMC4177463

[B189] Toledo-Arana A. Dussurget O. Nikitas G. Sesto N. Guet-Revillet H. Balestrino D.. (2009). The *Listeria* transcriptional landscape from saprophytism to virulence. Nature 459, 950–956. 10.1038/nature08080 19448609

[B190] Toledo-Arana A. Repoila F. Cossart P. (2007). Small noncoding RNAs controlling pathogenesis. Curr. Opin. Microbiol. 10, 182–188. 10.1016/j.mib.2007.03.004 17383223

[B191] Tolker-Nielsen T. Høiby N. (2009). Extracellular DNA and F-actin as targets in antibiofilm Cystic Fibrosis therapy. Future Microbiol. 4, 645–647. 10.2217/fmb.09.38 19659420

[B192] Tomasini A. Francois P. Howden B. P. Fechter P. Romby P. Caldelari I. (2014). The importance of regulatory RNAs in *Staphylococcus aureus*. Infect. Genet. Evol. 21, 616–626. 10.1016/j.meegid.2013.11.016 24291227

[B193] Tomasini A. Moreau K. Chicher J. Geissmann T. Vandenesch F. Romby P.. (2017). The RNA targetome of *Staphylococcus aureus* non-coding RNA RsaA: impact on cell surface properties and defense mechanisms. Nucleic Acids Res. 45, 6746–6760. 10.1093/nar/gkx219 28379505 PMC5499838

[B194] Toska J. Ho B. T. Mekalanos J. J. (2018). Exopolysaccharide protects *Vibrio cholerae* from exogenous attacks by the type 6 secretion system. Proc. Natl. Acad. Sci. U.S.A. 115, 7997–8002. 10.1073/pnas.1808469115 30021850 PMC6077691

[B195] Trotonda M. P. Tamber S. Memmi G. Cheung A. L. (2008). MgrA represses biofilm formation in *Staphylococcus aureus*. Infect. Immun. 76, 5645–5654. 10.1128/IAI.00735-08 18852246 PMC2583562

[B196] Tseng B. S. Reichhardt C. Merrihew G. E. Araujo-Hernandez S. A. Harrison J. J. MacCoss M. J.. (2018). A biofilm matrix-associated protease inhibitor protects *Pseudomonas aeruginosa* from proteolytic attack. mBio 9, e00543–e00518. 10.1128/mBio.00543-18 29636440 PMC5893882

[B197] Unterholzner S. J. Poppenberger B. Rozhon W. (2013). Toxin-antitoxin systems: biology, identification, and application. Mob. Genet. Elements 3, e26219–e26219. 10.4161/mge.26219 24251069 PMC3827094

[B198] Updegrove T. B. Zhang A. Storz G. (2016). Hfq: the flexible RNA matchmaker. Curr. Opin. Microbiol. 30, 133–138. 10.1016/j.mib.2016.02.003 26907610 PMC4821791

[B199] Vakulskas C. A. Potts A. H. Babitzke P. Ahmer B. M. M. Romeo T. (2015). Regulation of bacterial virulence by Csr (Rsm) systems. Microbiol. Mol. Biol. Rev. 79, 193–224. 10.1128/MMBR.00052-14 25833324 PMC4394879

[B200] Ventola C. L. (2015). The antibiotic resistance crisis: part 1: causes and threats. P T 40, 277–283. 10.1007/978-1-4614-6435-8_102103-1 25859123 PMC4378521

[B201] Wang L. Ruan S. (2017). Modeling nosocomial infections of Methicillin-Resistant *Staphylococcus aureus* with environment contamination. Sci. Rep. 7, 580–580. 10.1038/s41598-017-00261-1 28373644 PMC5428062

[B202] Wassarman K. M. (2007). 6S RNA: a small RNA regulator of transcription. Curr. Opin. Microbiol. 10, 164–168. 10.1016/j.mib.2007.03.008 17383220

[B203] Waters L. S. Storz G. (2009). Regulatory RNAs in bacteria. Cell 136, 615–628. 10.1016/j.cell.2009.01.043 19239884 PMC3132550

[B204] Wei Q. Ma L. Z. (2013). Biofilm matrix and its regulation in *Pseudomonas aeruginosa*. Int. J. Mol. Sci. 14, 20983–21005. 10.3390/ijms141020983 24145749 PMC3821654

[B205] Wen Y. Feng J. Sachs G. (2013). *Helicobacter pylori* 5′*ureB*-sRNA, a *cis*-encoded antisense small RNA, negatively regulates *ureAB* expression by transcription termination. J. Bacteriol. 195, 444–452. 10.1128/JB.01022-12 23104809 PMC3554021

[B206] Westermann A. J. (2018). Regulatory RNAs in virulence and host-microbe interactions. Microbiol. Spectr. 6:RWR-0002-2017. 10.1128/microbiolspec.RWR-0002-2017 30003867 PMC11633609

[B207] Wilking J. N. Zaburdaev V. De Volder M. Losick R. Brenner M. P. Weitz D. A. (2013). Liquid transport facilitated by channels in *Bacillus subtilis* biofilms. Proc. Natl. Acad. Sci. U.S.A. 110, 848–852. 10.1073/pnas.1216376110 23271809 PMC3549102

[B208] Williams McMackin E. A. Djapgne L. Corley J. M. Yahr T. L. (2019). Fitting pieces into the puzzle of *Pseudomonas aeruginosa* type III secretion system gene expression. J. Bacteriol. 201:e00209-19. 10.1128/JB.00209-19 31010903 PMC6560140

[B209] Winkler W. C. Breaker R. R. (2005). Regulation of bacterial gene expression by riboswitches. Annu. Rev. Microbiol. 59, 487–517. 10.1146/annurev.micro.59.030804.121336 16153177

[B210] Winstanley C. O'Brien S. Brockhurst M. A. (2016). *Pseudomonas aeruginosa* evolutionary adaptation and diversification in Cystic Fibrosis chronic lung infections. Trends Microbiol. 24, 327–337. 10.1016/j.tim.2016.01.008 26946977 PMC4854172

[B211] Wroblewski L. E. Peek R. M. Jr. Wilson K. T. (2010). *Helicobacter pylori* and gastric cancer: factors that modulate disease risk. Clin. Microbiol. Rev. 23, 713–739. 10.1128/CMR.00011-10 20930071 PMC2952980

[B212] Zhang Y.-F. Han K. Chandler C. E. Tjaden B. Ernst R. K. Lory S. (2017). Probing the sRNA regulatory landscape of *P. aeruginosa*: post-transcriptional control of determinants of pathogenicity and antibiotic susceptibility. Mol. Microbiol. 106, 919–937. 10.1111/mmi.13857 28976035 PMC5738928

[B213] Zimmermann A. Reimmann C. Galimand M. Haas D. (1991). Anaerobic growth and cyanide synthesis of *Pseudomonas aeruginosa* depend on *anr*, a regulatory gene homologous with *fnr* of *Escherichia coli*. Mol. Microbiol. 5, 1483–1490. 10.1111/j.1365-2958.1991.tb00794.x 1787798

